# Sexually Transmitted Infections and Male Circumcision: A Systematic Review and Meta-Analysis

**DOI:** 10.1155/2013/109846

**Published:** 2013-04-16

**Authors:** Robert S. Van Howe

**Affiliations:** Department of Pediatrics and Human Development, Michigan State University College of Human Medicine, 413 E. Ohio Street, Marquette, MI 49855, USA

## Abstract

The claim that circumcision reduces the risk of sexually transmitted infections has been repeated so frequently that many believe it is true. A systematic review and meta-analyses were performed on studies of genital discharge syndrome versus genital ulcerative disease, genital discharge syndrome, nonspecific urethritis, gonorrhea, chlamydia, genital ulcerative disease, chancroid, syphilis, herpes simplex virus, human papillomavirus, and contracting a sexually transmitted infection of any type. Chlamydia, gonorrhea, genital herpes, and human papillomavirus are not significantly impacted by circumcision. Syphilis showed mixed results with studies of prevalence suggesting intact men were at great risk and studies of incidence suggesting the opposite. Intact men appear to be of greater risk for genital ulcerative disease while at lower risk for genital discharge syndrome, nonspecific urethritis, genital warts, and the overall risk of any sexually transmitted infection. In studies of general populations, there is no clear or consistent positive impact of circumcision on the risk of individual sexually transmitted infections. Consequently, the prevention of sexually transmitted infections cannot rationally be interpreted as a benefit of circumcision, and any policy of circumcision for the general population to prevent sexually transmitted infections is not supported by the evidence in the medical literature.

## 1. Background

The earliest report of circumcision status as potential risk factor for sexually transmitted infections (STIs) was published in 1855 by Hutchinson, who noted that in men who were treated for STIs (primarily gonorrhea and syphilis), Jews were less likely to have syphilis [1]. This report is still referenced by circumcision proponents as a validation of their claim that circumcision prevents STIs, but the converse of Hutchinson's finding, namely that when compared to Gentiles, Jews were at greater risk for gonorrhea, is typically ignored.

The claim of reduction of the risk of STIs to justify neonatal circumcision continues today, often supported by selective bibliographies [2–12]. When the entire medical literature is reviewed, these claims become difficult to substantiate. The American Academy of Pediatrics, 1999, Task Force on Circumcision concluded that “evidence regarding the relationship of circumcision to STD in general is complex and conflicting.” [13] In 2012, using a selective bibliography, consistent with the practices of circumcision proponents, the American Academy of Pediatrics concluded that “evaluation of current evidence indicates that the health benefits of newborn male circumcision outweigh the risks; furthermore, the benefits of newborn male circumcision justify access to this procedure for families who choose it. Specific benefits from male circumcision were identified for the prevention of urinary tract infections, acquisition of HIV, transmission of some STIs, and penile cancer.” [12] Within the body of the statement, the committee admitted that they were unable to precisely measure the benefits of infant circumcision and unable to quantify the risks. The committee completed its review of the medical literature in April 2010 and published its findings in August 2012.

To shed some light on this contentious issue and whether the conclusion reached by the committee reflects the information available in the medical literature, this paper will provide a systematic review of the association between male circumcision status and the risk for individual types of STIs (other than human immunodeficiency virus (HIV)) and the overall risk for any STI. While a number of the review articles and systematic reviews of the association between male circumcision and individual types of STIs have been published [14–21], many of these need updating, while other have methodological shortcomings. This is also the first systematic review to explore the overall risk of contracting any STI.

## 2. Methods

The recommendations of Stroup et al. for the meta-analysis of observational studies were followed [22]. Articles were identified using a MEDLINE search and a review of references in published articles. A MEDLINE search using PubMed was undertaken on December 3, 2012. “Circumcision” was used as a key word, which identified 5472 articles. Inclusion criteria included cohort studies, cross-sectional studies, and case-control studies. The individual STIs included genital discharge syndrome (identified in studies as a generic term for gonorrhea, genital infections with *Chlamydia trachomatis*, and nonspecific (nongonococcal) urethritis in which the primary symptom was a urethral discharge) versus genital ulcerative disease (identified in studies as a generic term for syphilis, genital herpes, chancroid, and other genital ulcers noted on physical examination), genital discharge syndrome (GDS), nonspecific or nongonococcal urethritis (NSU), gonorrhea, genital infections with *Chlamydia trachomatis*, genital ulcerative disease (GUD), chancroid, syphilis, genital herpes or serology for herpes simplex virus type 2 (HSV), genital human papillomavirus (HPV) infections, and an STI of any type. Studies were also identified by reviewing references in published articles. For inclusion, publications needed to be in a peer-reviewed journal or government publication and present data on the circumcision status of males both with and without a specific STI or an STI in general. Studies primarily of men having sex with men or HIV-infected men were excluded. Within a study, identifiable men having sex with men and HIV-infected men were excluded from analysis, while heterosexual and HIV-negative men were included.

Articles meeting the inclusion criteria were read to determine the number of circumcised men with the illness, the number of circumcised men without the illness, the number of intact men with the illness, and the number of intact men without the illness. The primary analysis was performed using raw data, when available, for the published studies. In some cases, the raw data were obtained through back calculation with the information available in the article. Where raw data were not available, reported odds ratios, relative risks, and confidence intervals were used.

When distinct strata of the subjects within a study showed differing outcomes, each strata were considered separately in calculating the summary effect.

When data from the same population were published in one or more publications, the study in which the data reported the outcome of interest as a primary result or the most recent report were used.

Analyses of studies assessing disease incidence were conducted separately from studies of disease prevalence.

The impact of the type of study population was determined by separating the studies into those studying high-risk populations, such as attendees of sexually transmitted disease clinics and long-distance truck drivers in Africa, and those studying general populations. The impact of circumcision prevalence in the study population on the association between circumcision status and the prevalence of the various STIs was assessed using meta-regression.

Several studies meeting the inclusion criteria contained obvious forms of differential bias. A number of methods were employed to minimize the bias. Several older studies had inappropriate control groups [23–25]. For example, Hand used men without any exposure to STIs as controls [25]. In an attempt to control for exposure to STIs, men with a particular STI were compared to all men presenting for evaluation for the possibility of an STI.

The three randomized clinical trials of adult male circumcision in Africa failed to adjust for lead-time bias. Men in these trials who were assigned to immediate circumcision were instructed to either not engage in sexual activity or use condoms with all sexual contacts until the circumcision healed (approximately, from 4 to 6 weeks). Analyses that included these trials were conducted with the reported data and with the data adjusted for a six-week lead-time bias.

Other adjustments were needed specifically for the studies of HPV. Studies of the prevalence of genital HPV infections were separated into those identifying clinical infections with genital warts and those with diagnosis by culture, serology, biopsy, or polymerase chain reaction. Several studies reported separate data for all HPV infections and for infections with high-risk HPV that are potentially oncogenic. Consequently, two separate analyses were run on the latter group. In both analyses, the data from studies reporting only one set of data were used. In the first analysis, the data on all HPV infections were used, while the second analysis used the data on infections with high-risk HPV.

Previous analyses have found that the studies of HPV were prone to two forms of bias [16, 26–28]. The first was sampling bias. Several studies have found that circumcised men are more likely to have genital warts or have positive lesions or positive swabs on the penile shaft than intact men [29–35]. Consequently, studies that sampled only the glans or the urethra would underestimate the incidence and prevalence of HPV infection in circumcised males.

For example, in the study published by VanBuskirk et al., if only the glans is sampled, only 66.1% of the intact men with genital HPV would be identified, while only 45.2% of the circumcised men with genital HPV would be identified [32]. To adjust for the impact of this sampling bias, separate analyses were performed by multiplying the number of infections identified in studies that only sampled the glans by 1.514 in intact males and 2.212 in circumcised males.

The second is misclassification bias. Studies that rely on the patient report of circumcision status can often inaccurately identify the circumcision status of the participants. This has also been found to be a significant factor in previous analyses of HPV infections [16, 27, 28]. Finally, a separate analysis was conducted of studies of the prevalence of high-risk HPV in which the circumcision status of males was determined by physical examination and HPV was diagnosed by either serology or culture, biopsy, or polymerase chain reaction, with multiple site sampling including the shaft of the penis.

In one study, two testing methods for syphilis were used: the RPR results were used in this analysis [36].

### 2.1. Statistical Methods

For studies of disease prevalence, a general variance-based random-effects model was performed using each study's exact odds ratios (Proc-LogXact, version 5.0, Cytel Software Corporation, Cambridge, MA) as described previously [16]. DerSimonian and Laird random-effects summary results and between-study heterogeneity were calculated using the general variance-based method [37].

Poisson regression was used to assess studies of disease incidence. Fixed-effects summary results were calculated using Poisson regression. If between-study heterogeneity was significant (*P* < .10), random-effects summary results were calculated using the general variance-based method [37].

Sensitivity analyses of prevalence data for type of study population were performed through separate analyses for each population type. The impact of the type of study population, performance of a study in Africa, the prevalence of circumcision in the study population, and, for HPV, the sampling only the glans of the penis and determination of circumcision by physical examination was estimated using meta-regression [38].

To test for potential outliers, the dataset from each publication was individually excluded from the analysis to measure the impact on the chi-square measure of between-study heterogeneity. The exclusion of a study would be justified by a reduction of the between-study heterogeneity chi-square by a statistically significant amount (e.g., for one degree of freedom, a change in the chi-square value of more than 3.84). Sensitivity analysis was performed with each of these studies excluded and with the two most outlying studies excluded.

Publication bias was assessed using funnel graphs and linear regression analysis as described by Egger and associates [39], funnel plot regression as described by Macaskill et al. [40], and the adjusted rank correlation test described by Begg and Mazumdar [41]. Adjustment for publication bias was performed using the “trim and fill” method described by Duvall and Tweedie [42, 43]. Poisson regression and meta-regression were performed using SAS version 8.02 (SAS Institute, Cary, NC). 

## 3. Results

### 3.1. Search Results

The MEDLINE search identified 91 articles meeting the inclusion criteria. Of these, several reported on redundant study populations [44–55]. Twenty-one studies were identified through searches of bibliographies [1, 23, 25, 56–73]. Several studies had collected the data that would have met the inclusion criteria but did not report their results in a manner to include them in the analyses [70, 74–81]. The study by Rakwar et al. deserves special comment [70]. While this study was focused primarily on HIV infections, it also collected data on circumcision status and the prevalence and the incidence of GUD, GDS, chlamydia, gonorrhea, syphilis, HSV, genital warts, and chancroid. It did not include the results of these diseases by circumcision status. In a meta-analysis by Weiss et al., this study's results for chancroid are reported, but the study's results for HSV and syphilis are not [15].

The characteristics of the studies included for analysis and the types of STIs they studied are listed in Table 1. There were five studies that compared prevalence rates of circumcision in those with GUD with those with GDS. In the study by Nasio et al., only men who were not HIV infected were included. There were ten studies that documented prevalence rates of GDS. There was one study that documented incidence rates of GDS [82]. Twelve studies documented the prevalence of NSU. Three studies addressed the incidence and fourteen studies addressed the prevalence of genital *Chlamydia trachomatis*. Of the studies addressing gonorrhea, three studies looked at incidence and twenty-two looked at the prevalence. Two studies looked at the incidence of GUD, while twelve looked at the prevalence. For syphilis, there were three studies looking at incidence and twenty-seven studies looking at prevalence. For HSV, four studies looked at incidence and twenty-seven at prevalence. All four studies of chancroid documented prevalence. Of the studies of genital HPV, fourteen documented the prevalence of visible genital warts, seven documented the incidence, and twenty-one documented the prevalence of HPV infections. Some studies have looked at clearance rates of HPV from the penis, but these were not part of this analysis [35, 55, 83, 84]. Four studies looked at the incidence of contraction of any STI versus no STI, and twenty looked at prevalence.

### 3.2. Meta-Analysis Results

The results of the analyses of incidence data are shown in Table 2. Of note, when adjusted for lead-time bias, no statistically significant differences were noted in GDS, gonorrhea, syphilis, or any STI. GUD was significantly more common in intact men. For chlamydia, HSV, and HPV, intact men were at higher risk, but when adjusted for lead-time bias, the differences were no longer statistically significant. There was no evidence of significant between-study heterogeneity for any of these analyses.

The results of the analyses of prevalence data are shown in Tables 3, 4, 5, 6, 7, 8, 9, 10, 11, 12, 13, and 14. All of the analyses showed significant between-study heterogeneity. Intact men were found to be at significantly greater risk for GUD versus GDS, GUD, syphilis, and any HPV, while at significantly lower risk for NSU and genital warts. No significant differences were seen for chlamydia, gonorrhea, HSV, chancroid, or high-risk HPV. There was a trend for intact men be a lower overall risk for an STI that was statistically significant when a clear outlier studies is removed [85].

### 3.3. Outliers

The results of testing an individual publication's impact on between-study heterogeneity are shown in Table 15. Identifying and excluding the two studies with greatest impact on between-study heterogeneity was able to bring the overall between-study heterogeneity to within an acceptable range (*P* > .10) for GUD versus GDS, GDS, chlamydia, GUD, chancroid, and HPV but not NSU, gonorrhea, syphilis, HSV, genital warts, or any STI. Exclusion of studies did not change the conclusions of summary effect with only a few exceptions. In the analysis of genital warts, the removal of either the study by Oriel [29] or Wilson [23] made the negative association between intact men and genital warts statistically significant. A similar impact was seen in with HPV. In the analysis of any type of HPV, exclusion of the study by Vaccarella et al. brought the between-study heterogeneity within an acceptable range [86]. In the analysis of the prevalence of chancroid, exclusion of the study by Hart [69] brought the between-study heterogeneity to within an acceptable range and reversed the trend in the association. The most notable outlier was in the analysis of any STI, where the exclusion of the study by Langeni [85] resulted in a drop in the between-study heterogeneity chi-square of 203.41 (*P* < .0001) from 303.00 to 99.59. Consequently, two analyses of the prevalence of any sexual transmitted infections were conducted: one with and one without this study.

### 3.4. Sensitivity Analysis

Sensitivity analyses were not performed for the evaluation of risk of GUD versus GDS or chancroid because of the small number of studies. Sensitivity analysis comparing disease prevalence in studies of high-risk populations and general population is shown in Table 16. Of note, the association between intact men and the various STIs was consistently stronger in studies of high-risk populations. Intact men in general populations were at statistically significant lower risk of disease for GDS, NSU, genital warts, and any STIs with Langeni [85] excluded and at no statistically significant difference of risk for chlamydia, gonorrhea, syphilis, HSV, HPV, and any STIs with Langeni [85] included. Intact men were at greater risk of GUD in both general and high-risk populations. In high-risk populations, intact men were at significantly greater risk for GDS and syphilis and at no significant difference in risk for NSU, Chlamydia, gonorrhea, HSV, genital warts, HPV, or any STI. Between-study heterogeneity was within an acceptable range (*P* < .10) for high-risk populations for GDS and chlamydia and for general populations for gonorrhea and genital warts.

### 3.5. Meta-Regression Analysis

Meta-regression was not performed for the evaluation of risk of GUD versus GDS, chancroid, or the studies of disease incidence because of the small number of studies.

#### 3.5.1. High-Risk versus General Populations

Meta-regression methods found that the population type (general versus high risk) was notable (*P* < .10) for studies assessing the prevalence of GDS (*t* = 2.59 and  *P* = .0096), NSU (*t* = 1.79 and  *P* = .0735), and GUD (*t* = 1.67 and *P* = .0949). For the GDS studies, the summary effects were OR = 0.78 (95% CI = 0.62–0.96) for the general populations and OR = 1.11 (95% CI = 0.87–1.40) for the high-risk populations. For the NSU studies, the summary effects were OR = 0.61 (95% CI = 0.43–0.85) and OR = 0.85 (95% CI = 0.67–1.09) and for GUD 1.37 (95% CI = 1.00–1.85) and (95% CI = 1.50–2.10) for general and high-risk populations, respectively.

No significant differences were seen for chlamydia, gonorrhea, syphilis, HSV, genital warts, HPV, or any STI (either with or without the study by Langeni [85] was included).

#### 3.5.2. Studies in Africa

Meta-regression methods found that having a study carried out in Africa as opposed to elsewhere was notable (*P* < .10) for studies assessing the prevalence of GDS (*t* = −1.77 and *P* = .0767), chlamydia (*t* = −1.71 and *P* = .0873), GUD (*t* = −3.38 and *P* = .0009), and any type of HPV (*t* = 1.68 and *P* = .0930). For GDS, the summary odds ration in Africa was 0.85 (95% CI = 0.70–0.97) and 1.19 (95% CI = 0.80–1.78) outside Africa. For chlamydia, the summary odds ratio in Africa was 0.63 (95% CI = 0.35–1.12), while it was 1.0098 (95% CI = 0.85–1.21) outside of Africa. For GUD, the summary odds ratio inside Africa was 1.45 (95% CI = 1.24–1.70), while it was 1.95 (95% CI = 1.74–2.18) outside of Africa. For any HPV type, the summary odds ratios are 2.13 (95% CI = 1.05–4.29) and 1.18 (95% CI = 0.9919–1.41) inside and outside of Africa, respectively. The studies of HSV showed a trend to having a greater association between HSV and intact men inside of Africa (*t* = 1.53 and *P* = .1261) with African studies having a summary odds ratio of 1.35 (95% CI = 1.04–1.75) and non-African studies a summary odds ratio of 1.06 (95% CI = 0.87–1.29).

No significant difference was seen in with NSU, gonorrhea, syphilis, genital warts, high-risk HPV, or any STI.

#### 3.5.3. Circumcision Prevalence

**Figure 1:**
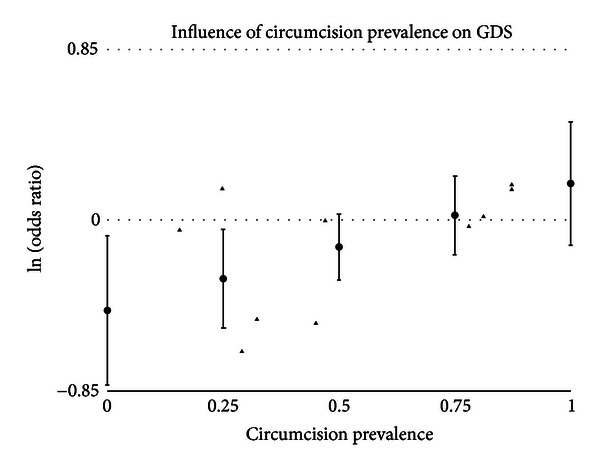
Natural logarithm of odds ratio as a function of the prevalence of circumcision in the population when estimating the prevalence of genital discharge syndrome by circumcision status in adult men. Solid triangles represent individual populations. Circles represent estimates and 95% confidence intervals using meta-regression.

**Figure 2:**
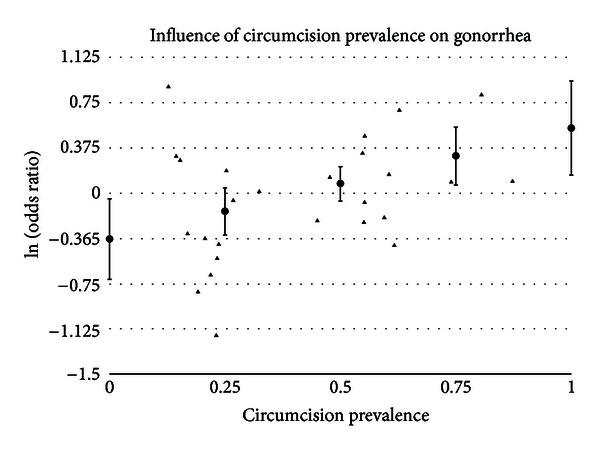
Natural logarithm of odds ratio as a function of the prevalence of circumcision in the population when estimating the prevalence of gonorrhea by circumcision status in adult men. Solid triangles represent individual populations. Circles represent estimates and 95% confidence intervals using meta-regression.

**Figure 3:**
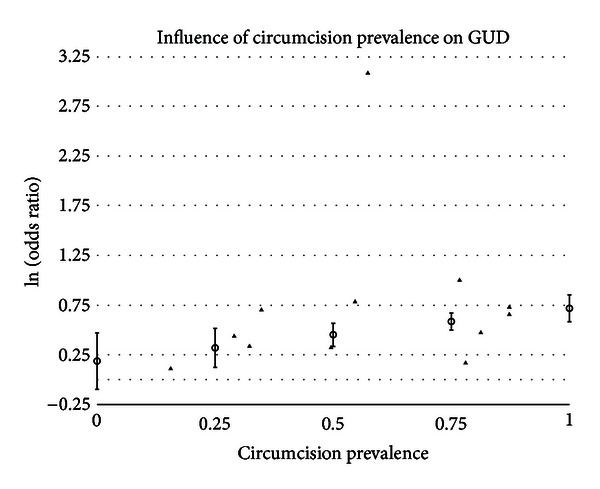
Natural logarithm of odds ratio as a function of the prevalence of circumcision in the population when estimating the prevalence of genital ulcerative disease by circumcision status in adult men. Solid triangles represent individual populations. Circles represent estimates and 95% confidence intervals using meta-regression.

**Figure 4:**
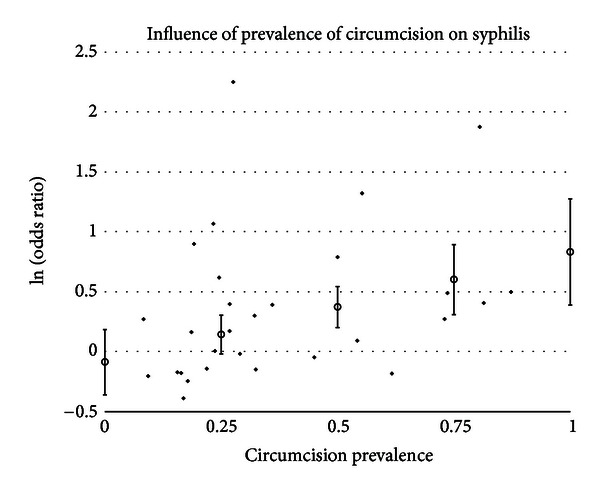
Natural logarithm of odds ratio as a function of the prevalence of circumcision in the population when estimating the prevalence of syphilis by circumcision status in adult men. Solid triangles represent individual populations. Circles represent estimates and 95% confidence intervals using meta-regression.

**Figure 5:**
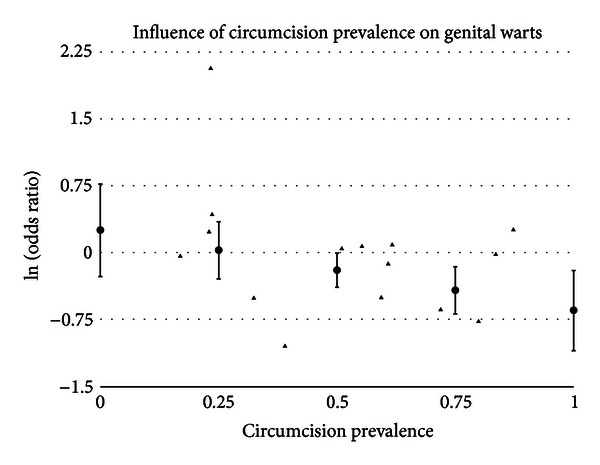
Natural logarithm of odds ratio as a function of the prevalence of circumcision in the population when estimating the prevalence of genital warts by circumcision status in adult men. Solid triangles represent individual populations. Circles represent estimates and 95% confidence intervals using meta-regression.

A statistically significant impact of circumcision prevalence on the natural logarithm of the odds ratio of the association between circumcision status and prevalence of disease was found for GDS (*t* = 2.43 and *P* = .0151), gonorrhea (*t* = 2.82 and *P* = .0048), GUD (*t* = 3.09 and *P* = .0020), syphilis (*t* = 2.86 and *P* = .0042), and genital warts (*t* = −2.14 and *P* = .0324). The impact of circumcision prevalence on disease risk is shown in Figures 1–5. The odds ratios increased with circumcision prevalence for all diseases, except for the opposite association with genital warts. Circumcision prevalence was not a statistically significant factor for the other diseases.

#### 3.5.4. Combinations of Factors

For GUD, population type, a study being performed in Africa, and circumcision prevalence were all statistically significant factors. When multiple factors are added to the regression model, only a study being performed in Africa was statistically significant. A model with a general population performed in Africa found a random effects summary odds ratio of 1.33 (95% CI = 1.02–1.71).

#### 3.5.5. Studies of HPV

With the studies of any type of HPV, sampling only the glans trended toward being a factor (*t* = 1.57 and *P* = .1165). Glans only studies had a summary odds ratio of 1.82 (95% CI = 1.05–3.14), while studies with complete sampling had a summary odds ratio of 1.17 (95% CI = 0.98–1.40). Patient report of circumcision status was a statistically significant factor (*t* = 2.26 and *P* = .0237) with studies relying on physical examination to determine circumcision status having a summary odds ratio of 1.14 (95% CI = 0.97–1.35) and studies with a reliance on patient report as summary odds ratio of 2.11 (95% CI = 1.24–3.59). When both factors are included in a multivariate model (sampling *t* = 1.91 and *P* = .0562; physical examination *t* = 2.53 and *P* = .0114), the summary odds ratio for complete sampling of the penis combined with circumcision status determined by physical examination is 1.08 (95% CI = 0.93–1.24), and for sampling only the glans combined with determining circumcision status by patient report is 3.21 (95% CI = 1.62–6.36).

With high-risk HPV studies, sampling only the glans trended toward being a factor (*t* = 1.64 and *P* = .1011). Studies that sample only the glans had a summary odds ratio of 1.86 (95% CI = 0.9964–3.46), while studies with complete sampling had a summary odds ratio of 1.10 (95% CI = 0.88–1.37). Patient report of circumcision status was statistically significant (*t* = 2.24 and *P* = .0251) with physical examination studies having a summary odds ratio of 1.08 (95% CI = 0.88–1.32) and patient report studies having a summary odds ratio of 2.16 (95% CI = 1.18–3.99). When both factors are included in model (sampling *t* = 1.92 and *P* = .0549; physical examination *t* = 2.47 and *P* = .0135), the summary odds ratio for complete sampling combined with physical examination determination of circumcision status is 1.01 (95% CI = 0.84–1.22), while the summary odds ratio with sampling only the glans combined with depending on patient report to determine circumcision status is 3.45 (95% CI = 1.60–7.42).

### 3.6. Publication Bias

**Figure 6:**
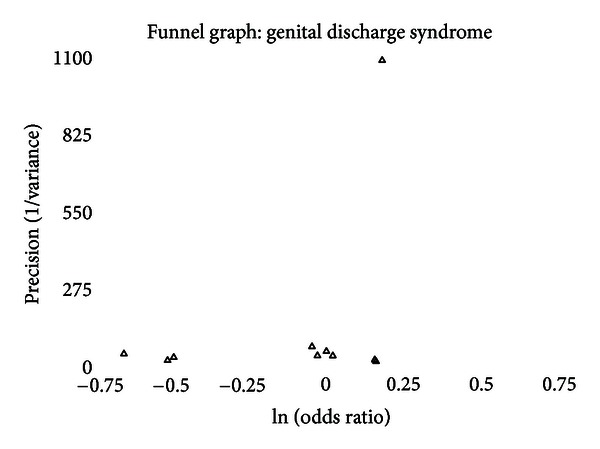
Funnel graph of precision (1/variance) by the natural logarithm of the odds ratio of studies estimating the prevalence of genital discharge syndrome by circumcision status in adult men. Empty triangles represent published studies.

**Figure 7:**
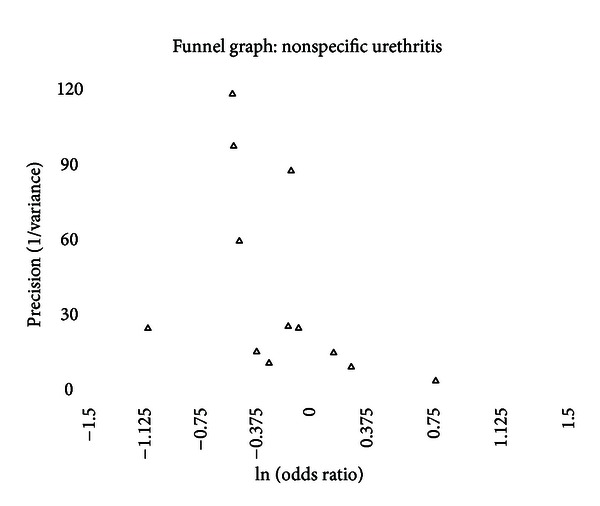
Funnel graph of precision (1/variance) by the natural logarithm of the odds ratio of studies estimating the prevalence of nonspecific (nongonococcal) urethritis by circumcision status in adult men. Empty triangles represent published studies.

**Figure 8:**
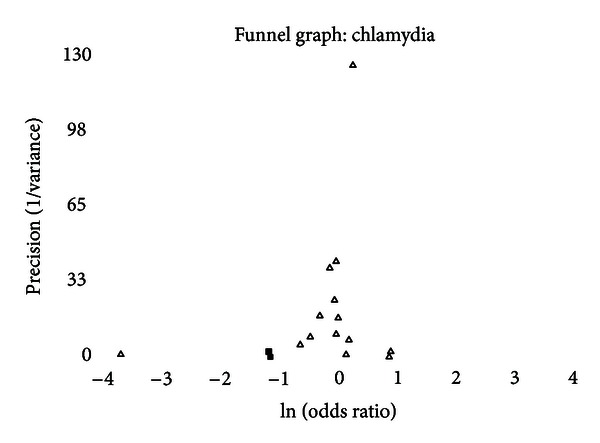
Funnel graph of precision (1/variance) by the natural logarithm of the odds ratio of studies estimating the prevalence of genital infections with *Chlamydia trachomatis* by circumcision status in adult men. Solid squares represent likely unpublished studies using the “trim and fill” method.

**Figure 9:**
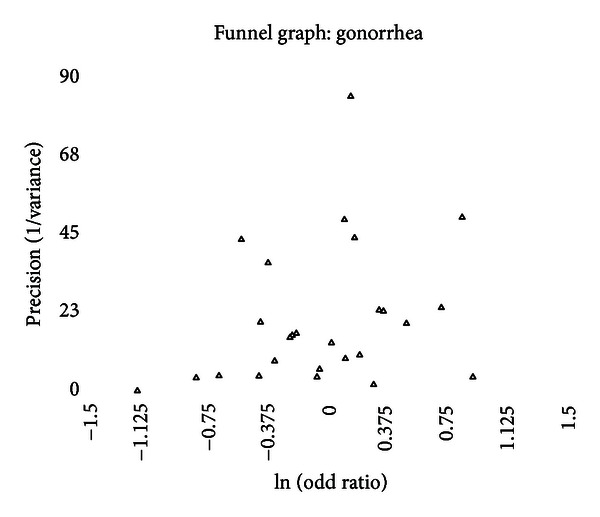
Funnel graph of precision (1/variance) by the natural logarithm of the odds ratio of studies estimating the prevalence of gonorrhea by circumcision status in adult men. Empty triangles represent published studies.

**Figure 10:**
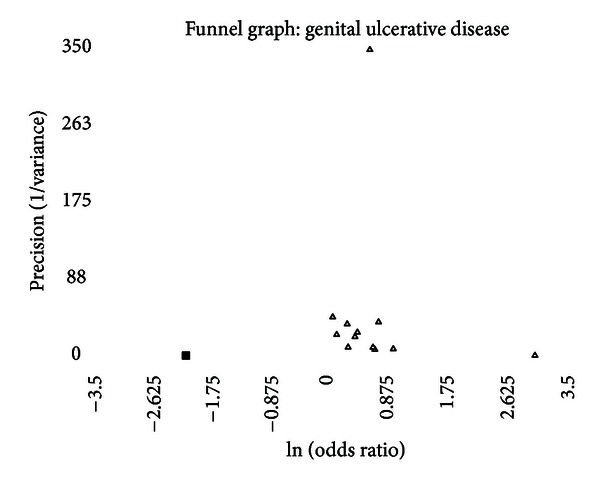
Funnel graph of precision (1/variance) by the natural logarithm of the odds ratio of studies estimating the prevalence of genital ulcerative disease by circumcision status in adult men. Empty triangles represent published studies. Solid squares represent likely unpublished studies using the “trim and fill” method.

**Figure 11:**
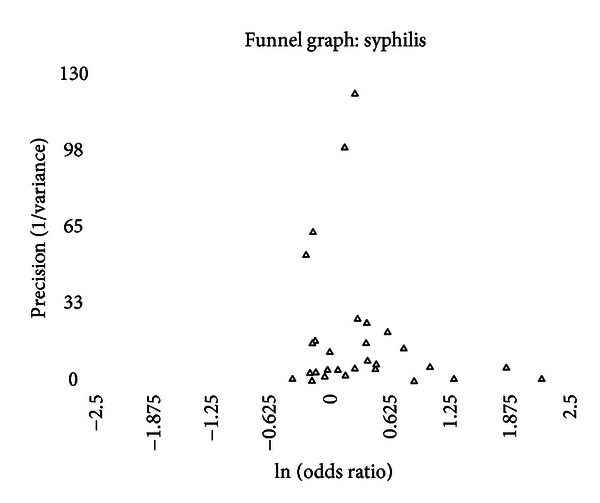
Funnel graph of precision (1/variance) by the natural logarithm of the odds ratio of studies estimating the prevalence of syphilis by circumcision status in adult men. Empty triangles represent published studies.

**Figure 12:**
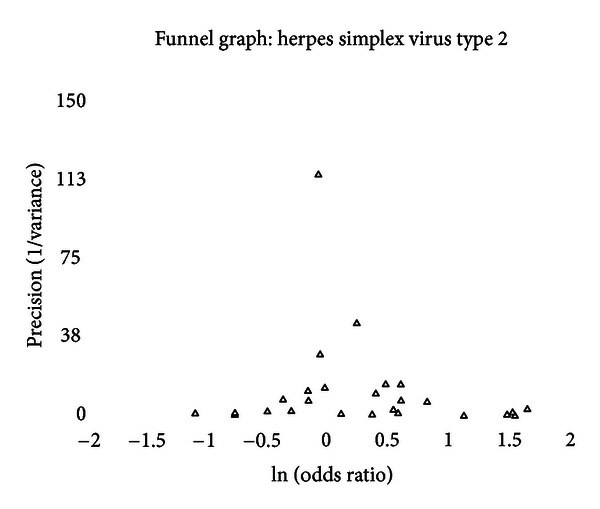
Funnel graph of precision (1/variance) by the natural logarithm of the odds ratio of studies estimating the prevalence of genital herpes/herpes simplex virus type 2 by circumcision status in adult men. Empty triangles represent published studies.

**Figure 13:**
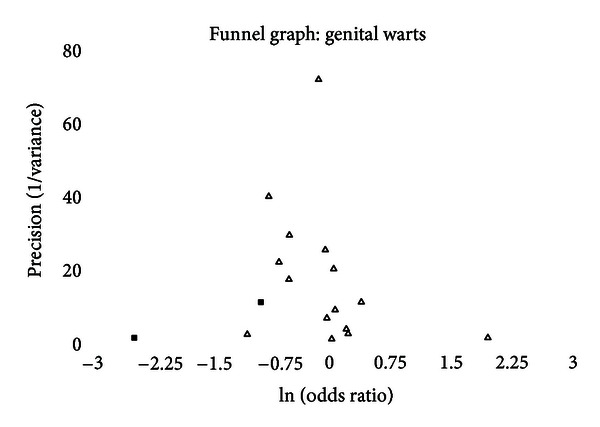
Funnel graph of precision (1/variance) by the natural logarithm of the odds ratio of studies estimating the prevalence of genital warts by circumcision status in adult men. Empty triangles represent published studies. Solid squares represent likely unpublished studies using the “trim and fill” method.

**Figure 14:**
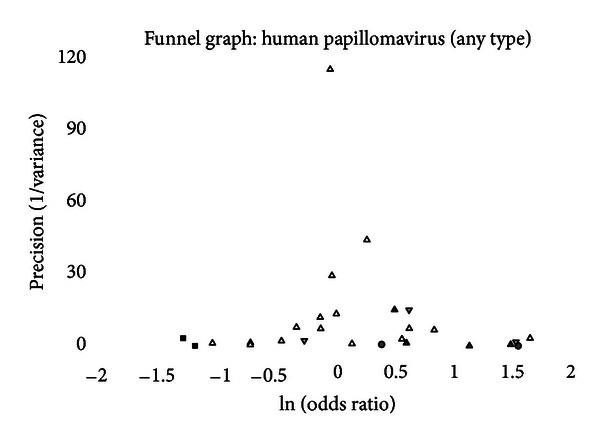
Funnel graph of precision (1/variance) by the natural logarithm of the odds ratio of studies estimating the prevalence of genital human papillomavirus of any type by circumcision status in adult men. Empty triangles represent published studies in which the entire penis was sampled and circumcision status was determined by physical examination. Shaded triangles represent published studies in which only the glans was sampled and circumcision status was determined by physical examination. Inverted shaded triangles represent published studies in which entire penis was sampled and circumcision status was determined by patient report. Shaded circles represent published studies in which only the glans was sampled and circumcision status was determined by patient report. Solid squares represent likely unpublished studies using the “trim and fill” method.

**Figure 15:**
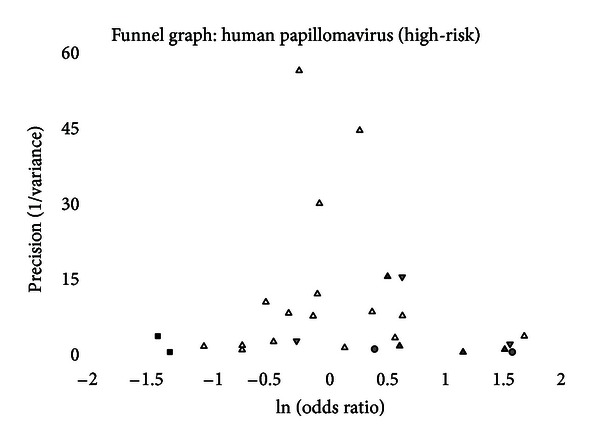
Funnel graph of precision (1/variance) by the natural logarithm of the odds ratio of studies estimating the prevalence of genital human papillomavirus of focussing on those at high-risk oncogenic potential by circumcision status in adult men. Empty triangles represent published studies in which the entire penis was sampled and circumcision status was determined by physical examination. Shaded triangles represent published studies in which only the glans was sampled and circumcision status was determined by physical examination. Inverted shaded triangles represent published studies in which entire penis was sampled and circumcision status was determined by patient report. Shaded circles represent published studies in which only the glans was sampled and circumcision status was determined by patient report. Solid squares represent likely unpublished studies using the “trim and fill” method.

**Figure 16:**
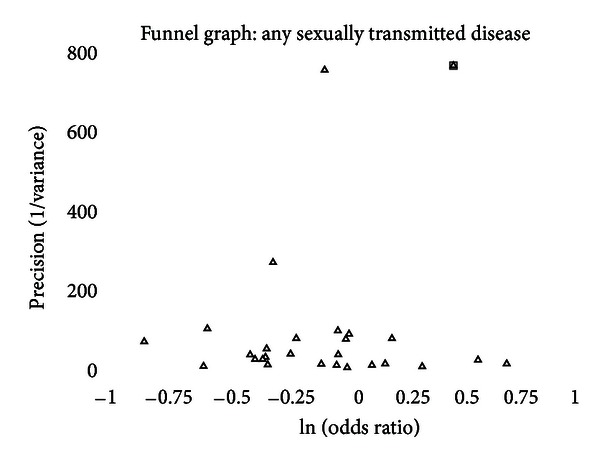
Funnel graph of precision (1/variance) by the natural logarithm of the odds ratio of studies estimating the prevalence of any sexually transmitted infection versus no sexually transmitted infection by circumcision status in adult men. Empty triangles represent published studies. An outlier study [85] is identified by an open square.

A funnel graph, which plots the precision (the inverse of variance) on the *y*-axis and the natural logarithm of the odds ratio on the *x*-axis, should have a shape like an inverted funnel with the largest study representing the apex of the inverted funnel. If there is a paucity of studies in the left lower portion of the inverted funnel and a cluster of studies in the right lower portion, that would be suggestive of publication bias. Funnel graphs for the various STIs are shown in Figures 6–16. Paucity in the left lower portion is seen in the funnel graphs for NSU (Figure 7), GUD (Figure 10), syphilis (Figure 11), genital warts (Figure 13), and HPV (Figures 14 and 15). Large studies that appear to be outliers (odds ratios greater than expected) are noted in funnel graphs for GDS (Figure 6) [87], HSV (Figure 12) [88], and any STI (Figure 16) [85]. The funnel graph for chlamydia shows an outlier in left lower portion (Figure 8) [89].

Methods to determine the presence of publication bias use a *P* value threshold of 0.10 for significance. Results of evaluation for publication bias using for each STI are shown in Table 17. Of the six measures of publication bias, none were positive for GUD, syphilis, and genital warts; one was positive for chlamydia, gonorrhea, HSV, and any STI with the study by Langeni [85] excluded; three were positive for NSU and HPV; and four were positive for GDS and any STI with Langeni [85] included.

#### 3.6.1. Trim and Fill

Using the “trim and fill” technique, no adjustments were needed for studies of the prevalence of GDS, NSU, gonorrhea, syphilis, HSV, HPV (in which there was complete sampling and circumcision status that was determined by physical examination), and any STI.

For genital infections with chlamydia, the “trim and fill” technique indicated two unpublished studies. By adding these two studies, the summary odds ratio, adjusted for publication bias, is 0.88 (95% CI = 0.69–1.11). The addition of one study was indicated for GUD, the addition of which yield a summary odds ratio, adjusted for publication, of 1.64 (95% CI = 1.34–2.01). For genital warts, the technique indicated two unpublished studies, whose addition would yield a summary odds ratio, adjusted for publication bias, of 0.76 (95% CI = 0.60–0.97). For both analyses of the prevalence of HPV infections (any type and high-risk types), two unpublished studies would be expected. The summary odds ratio, adjusted for publication, would be 1.19 (95% CI = 0.97–1.46) for any HPV types and 1.11 (95% CI = 0.88–1.39) for using high-risk HPV types.

## 4. Discussion

### 4.1. Genital Ulcerative Disease versus Genital Discharge Syndrome

The comparisons of men diagnosed with GUD and GDS are consistent with findings that intact men are more prone to GUD and circumcised men are more prone to GDS. Consequently, there is no surprise here.

### 4.2. Genital Discharge Syndrome

The prevalence of GDS shows a moderate trend toward being less common in intact men (OR = 0.89 and 95% CI = 0.73–1.09). The finding in general populations is statistically significant (OR = 0.77 and 95% CI = 0.59–0.99). The only study of incidence found no significant difference [82]. Circumcision prevalence in the population studied had a significant association with the odds ratio measured for prevalence of GDS (Figure 1). The funnel graph (Figure 6) indicates that the study by Warner et al., [87] to be an outlier. This is confirmed when this study is excluded from the analysis and the summary odds ratio drops to 0.85 and the finding approaches statistical significance (95% CI = 0.70–1.03) (Table 15). This study also may explain why four of the measures of publication bias were positive. While this diagnosis is based on clinical findings, the lack of association with intact men and GDS is consistent with what is seen with NSU.

### 4.3. Nonspecific (Nongonococcal) Urethritis

The prevalence of NSU is significantly lower in intact males (OR = 0.76 and 95% CI = 0.63–0.92). Between-study heterogeneity is a concern as five of the twelve studies contributed significantly to the between-study heterogeneity, but exclusion of any these studies did not change the significance of this finding (Table 15). Three publication bias measures were positive, which is consistent with the paucity of studies in lower left portion of the funnel graph (Figure 7). The “trim and fill” method, however, found that no studies were needed to adjust for publication bias. 

Other than the problems with between-study heterogeneity and these analysis indicates a fairly robust, significant association between a lower prevalence of NSU in intact males.

### 4.4. Chlamydia

There was no significance difference in the prevalence of genital chlamydia infections but a trend toward a lower prevalence in intact men. None of the studies of incidence found a significant difference (whether adjusted for lead-time bias or not). When studies of incidence are adjusted for lead-time bias and combined, there is no significant association.

Only two outliers were identified (Table 15). When they are excluded from the analysis, the summary odds ratio is 0.93 (95% CI = 0.87–1.00) and the between-study heterogeneity resolves (chi-square = 7.75 (df = 11) and *P* = .7357). Meta-regression showed a trend toward a lower association between the prevalence of chlamydia and intact men in African studies (*P* = .0873). In African studies, the summary odds ratio was 0.63 (95% CI = 0.35–1.12).

The funnel graph indicates a clear outlier (Figure 8) [89]. Two of the measures of publication were positive, and the “trim and fill” method added two studies to the left lower portion of the graph, giving a summary odds ratio, adjusted for publication bias of 0.87 (95% CI = 0.69–1.11).

The analysis indicates a trend toward a lower prevalence of chlamydia in intact men, especially in Africa and in the general population. No difference was seen in the incidence studies.

### 4.5. Gonorrhea

No significant association between the incidence or the prevalence of gonorrhea and circumcision status of males was found. This was seen in both high-risk and general populations. There was significant between-study heterogeneity, and five potential outliers were identified. The prevalence of circumcision in the population studied was significantly associated with odds ratio reported in the study (*P* = .0048) (Figure 2). As circumcision prevalence approached the extremes, the summary odds ratio in population with a 0% circumcision rate would be estimated at 0.68 (95% CI = 0.49–0.96), while a population with a 100% circumcision rate, the summary odds ratio would be estimated at 1.72 (95% CI = 1.16–2.55).

Only one measure of publication bias was positive, and the funnel graph (Figure 9) looks symmetric. No studies were added using the “trim and fill” approach.

The data indicate that the incidence and the prevalence of gonorrhea are not affected by circumcision status as much as by the prevalence of circumcision within the community studied.

### 4.6. Genital Ulcerative Disease

Incidence and prevalence of GUD were consistently positively associated with intact men, even when subjected to sensitivity analysis and meta-regression. Between-study heterogeneity was significant even after adjusting for four “outlying” studies. Meta-regression found significant associations for population type, whether studies were performed in Africa and circumcision prevalence in the populations studied (Figure 3). When combined in a multivariate analysis, only a study being performed in Africa was a significant factor.

In the funnel graph, there is a study in the right lower portion that is not balanced in the left lower portion (Figure 10). None of the publication bias measures were positive, yet the “trim and fill” process added one study making summary odds ratio, adjusted for publication bias, of 1.63 (95% CI = 1.34–2.01).

GUD, which is more commonly seen in developing countries, has a propensity for mucosal surfaces. Most of the studies of HSV have looked at seroconversion rates for herpes simplex virus type 2. This will not capture recurrences. Since GUD is a clinical measure that includes HSV recurrences and ulcers for which no causative agent can be identified; one would expect a higher rate in intact men because more than half of the mucosal surface of the penis is removed with circumcision. Herpes simplex viruses, including type 1 and type 2, also have a propensity for junctional tissues. This is why cold sores recur in the corner of the mouth and on the facial lips. If one were to amputate facial lips, one would see a lower recurrence rate of herpes simplex virus type 1. To follow this analogy, circumcision removes all of the junctional tissue of the prepuce [90, 91], so this may impact HSV recurrences. While this is a consistent finding, it is difficult to know what the public health impact is in regions where the prevalence of GUD is low.

### 4.7. Syphilis

The data on syphilis present quite a farrago. On the one hand, there is a positive association between the prevalence of syphilis and intact genitalia, but, on the other hand, the incidence of syphilis, even before adjusting for lead-time bias, indicates a negative, albeit nonsignificant, association. The positive association is seen primarily in populations at high risk for acquiring STIs, while in the prevalence in general populations found no statistically significant difference (depending on the calculation method used such as general variance-based method: OR = 1.23 and 95% CI = 1.0064–1.49; meta-regression method: OR = 1.25 and 95% CI = 0.96–1.60). Seven prevalence studies had statistically significant contributions to the between-study heterogeneity. The between-study heterogeneity improves when only studies of general populations are considered but does not resolve completely. The prevalence of syphilis by circumcision status is also significantly associated with the prevalence of circumcision in the population studied (Figure 4).

The funnel graph clearly looks asymmetric (Figure 11), but none of the measures of publication bias nor the “trim and fill” method identified this.

With the mixed results between incidence and prevalence, the lack of a significant association in general populations, the number of studies that could be considered outliers, the significant association with circumcision prevalence in the population studied, and the asymmetry of the funnel graph, one cannot accurately conclude that the risk of syphilis is significantly associated with circumcision status.

### 4.8. Genital Herpes/Herpes Simplex Virus Type 2

While there was a trend for the prevalence of HSV to be greater in intact men, the association was not statistically significant. When adjusted for lead-time bias, none of the studies that looked at the incidence of herpes simplex virus type 2 found a statistically significant association. When the studies are combined, there is no statistically significant association but a slight trend toward higher risk for intact men.

There was significant between-study heterogeneity for the prevalence studies. Six outliers were identified. Exclusion of these studies individually and the two largest contributors did not bring the between-study heterogeneity within an acceptable range and did not yield a summary effect that was statistically significant. In both high-risk and general populations, the summary effect was not statistically significant, and between-study heterogeneity remained significant. Using meta-regression, there was a trend (*P* = .1261) that odds ratios were higher in African studies.

The funnel graph indicates some asymmetry with a cluster of studies in the lower right portion that is not balanced on the left side (Figure 12). Two of the measures of publication bias were positive, but no adjustments were indicated using the “trim and fill” method.

While there is a trend toward higher incidence and prevalence of HSV in intact men, the finding is persistently not statistically significant despite a number of adjustments. The high level of between-study heterogeneity, which could not be shed despite several attempts, presents a problem in making any recommendation regarding circumcision's impact on HSV.

An earlier meta-analysis of HSV prevalence and circumcision had failed to include two of the populations included in this analysis [15, 65]. This is strange considering that the same person was the lead author of both studies.

As an aside, there have been a number of systemic and fatal herpetic infections reported following ritual circumcision in which the person performing the circumcision puts his mouth around the penis after the foreskin has been amputated [92–95]. Instead of banning the practice, the New York City Health Department has asked parents to sign off on this practice. Orthodox Jews in New York City are currently fighting this ruling.

### 4.9. Chancroid

The paucity of studies, the reliance on clinical identification in all but one of these studies, and the high degree of between-study heterogeneity make it difficult to comment on the impact of circumcision on this illness, yet the lack of good evidence did not keep the 2012 AAP Task Force from including a discussion of circumcision's impact on the prevalence of chancroid [12], which is relatively uncommon in developing nations and extremely rare in developed nations. The degree of between-study heterogeneity is significant and can be almost completely attributed to one study [69]. Exclusion of this study brought the between-study heterogeneity within an acceptable range (*P* = .1128). When other outliers were excluded from analysis along with the study by Hart [69], the further reduction in the between-study heterogeneity chi-square, compared to excluding only Hart's study, was not statistically significant.

The data do not support the claim by Weiss et al. that “circumcised men are at lower risk of chancroid” [15]. There have been no new publications on the impact of circumcision on the prevalence of chancroid since 2006. The difference between the analyses is that Weiss et al. included several studies in their meta-analysis that were not strictly studies of chancroid. As I have noted previously [96], three of the studies included in their analysis of chancroid did not meet basic inclusion criteria because they lacked a direct comparison between intact and circumcision men for a specific diagnosis of chancroid [59, 97, 98]. In two of the studies, men with genital ulcers were *presumed* to have chancroid but never tested for it [97, 98], while the third study tested the men presumed to have chancroid and found that 31.4% had herpes simplex virus type 2 and only 22.9% had a positive culture for *Haemophilus ducreyi*, the causative agent of chancroid [59]. When these studies are appropriately assigned to an analysis of the prevalence of GUD and excluded from an analysis of the prevalence of chancroid, any imagined association between circumcision status and prevalence of chancroid evaporates.

### 4.10. Genital Warts

The prevalence of genital warts has a strong trend towards being lower in intact males. In general populations, the association is statistically significant (OR = 0.78 and 95% CI = 0.63–0.96) and did not have evidence of between-study heterogeneity (chi-square = 8.61 (df = 6) and *P* = .1969). Three studies were identified as potential outliers; removal of the two studies with the greatest impact on between-study heterogeneity brought the between-study heterogeneity near the acceptable range (*P* = .0901). Using meta-regression, circumcision prevalence in the population studied was negatively associated with the reported odds ratios (*P* = .0324) (Figure 5).

The funnel graph indicates some paucity of studies in the left lower region (Figure 13). None of the measures of publication bias were positive, yet the “trim and fill” calculations indicated that there were two studies missing in the left lower portion of the funnel graph. Adjusting for publication bias, the summary odds ratio was 0.76 (95% CI = 0.60–0.97).

The evidence in favor of a lower prevalence of genital warts in intact males is supported by the finding in studies of general populations, which were surprisingly free of between-study heterogeneity and the summary result after adjusting for publication bias. The odds ratios in studies were, however, impacted by the prevalence of circumcision in the population studied.

### 4.11. Human Papillomavirus

A systematic review of the incidence and prevalence of genital HPV infections as they relate to circumcision status in males is fraught with a variety of pitfalls. This may explain why several systematic reviews with meta-analysis have been published with inconsistent results [16, 18, 20]. HPV has many subtypes, some of which have been demonstrated to be oncogenic, while others are benign and self-limited infections. The oncogenic types have been strongly linked to cervical cancer in women and may be responsible for about half of the cases of penile cancer in men. Some studies reported their results for HPV infections without specifying the types of HPV identified, some reported only infections with oncogenic HPV, and some studies reported results on all HPV infections and also infections with oncogenic HPV. Consequently, two analyses were run (any HPV and high-risk HPV). Since oncogenic HPV is more concerning clinically, the second analysis may be the more relevant of the two. In the analysis that focused on high-risk HPV, there was no significant difference in the prevalence by circumcision status.

Previous analyses have found that sampling bias and patient report of circumcision status significantly effect the odds ratio reported in a study [16, 26–28]. For this reason, a third analysis (selective HPV) was run on the studies of prevalence in the second analysis (high-risk HPV) in which studies with the potential for sampling bias and misclassification bias were excluded.

Finally, the two randomized clinical trials that reported their results on HPV infection both failed to adjust for sampling only the glans and to adjust for lead-time bias.

The incidence of HPV infections was barely statistically significantly different based on circumcision status before adjustment for sampling bias and lead-time bias (RR = 1.16 and 95% CI = 1.0097–1.34). After adjustment for these sources of bias, the relative risk is 0.96 (95% CI = 0.85–1.09).

Prevalence of HPV in the first analysis (any HPV) was higher in intact men (OR = 1.24 and 95% CI = 1.02–1.50), but the statistical significance of this finding is tenuous. When sensitivity analysis comparing studies of high-risk populations and studies of general populations, the result in neither group is statistically significant. When two of the identified “outliers” are individually excluded from the analysis, the results are not statistically significant.

When meta-regression is used to adjust for sampling bias, and misclassification bias the summary odds ratio is 1.08 (95% CI = 0.93–1.24).

The funnel graph for the first analysis of HPV (any HPV) shows a clear paucity of studies in the left lower portion (Figure 14). Not surprisingly, three of the measures of publication bias were positive, and the “trim and fill” method added two studies. The summary odds ratio adjusting for publication bias was 1.19 (95% CI = 0.97–1.46).

Prevalence of HPV in the second analysis (high-risk HPV) was not significantly different on the basis of circumcision status (OR = 1.17 and 95% CI = 0.94–1.45). Significant difference was found in neither high-risk populations nor general populations.

Five outliers were identified. Excluding them individually from the analysis or excluding the two studies that contributed the most to between-study heterogeneity did not result in providing evidence of statistically significant difference. Excluding the two studies did bring between-study heterogeneity to within an acceptable range (*P* = .1303). The summary odds ratio with these studies excluded was 1.16 (95% CI = 0.95–1.41).

Using meta-regression to adjust for sampling bias and misclassification bias the summary odds ratio was 1.01 (95% CI = 0.84–1.22).

The funnel graph for the second analysis also shows a paucity in the left lower portion (Figure 15). Three measures of publication were positive, and “trim and fill” methods indicated the absence of two studies. The summary odds ratio, adjusting for publication bias, was 1.10 (95% CI = 0.88–1.39).

Prevalence of HPV in the third analysis (selective HPV) was nearly identical in intact and circumcised men (OR = 1.01 and 95% CI = 0.80–1.28). Three studies were identified as outliers. Exclusion of the study with the largest contribution to the between-study heterogeneity [86] resulted in the between-study heterogeneity coming with an acceptable range (*P* = .1689) and yielded a summary odds ratio of 0.96 (95% CI = 0.79–1.15). The funnel plot for the studies included in the third analysis was symmetrical, all measures of publication bias were negative, and no addition of studies were indicated by the “trim and fill” analysis.

There are several messages from the three analyses performed on the HPV prevalence studies. Sampling bias and misclassification bias have a significant differential effect on the odds ratios reported in studies where these forms of bias are suspected. There is no significant difference in the incidence or the prevalence of HPV (especially oncogenic HPV) on the basis of circumcision status. While circumcision proponents repeatedly laud circumcision as preventive for HPV infections, the data do not support this claim. When their own studies are adjusted for lead-time bias and sampling bias, their treatment effect disappears [26–28].

Several studies of HPV and circumcision status warrant additional comment because of their serious methodological flaws. One study compiled data collected from seven studies in five countries from three continents. A fatal flaw in the study was the small number of circumcised men in four of the countries and the small number of intact men in the fifth country. Of the twenty data cells that make up the two-by-two tables from the five countries, seven had five or fewer subjects. The authors used parametric statistical methods, which are notably unreliable in this situation, to report the statistics on the combined data [99]. Unfortunately, this study, which did not find a statistically significant association between circumcision status of male sexual partners and cervical cancer, has been quoted by circumcision proponents, including the authors of the study, as demonstrating that circumcision prevents cervical cancer. Given the problems with small number of men in many of the data cells described above, it would be impossible to accurately perform the subset results they reported for cervical cancer.

The study published by Lajous et al. is problematic in that fourteen men were identified as circumcised on physical examination, while 95 men identified themselves as being circumcised. Although physical examination is considered the gold standard for assigning circumcision status, instead of using physical examination as the measure of circumcision status, the study published the association between HPV infection and self-report of circumcision. Eighty-eight of the 95 men who reported themselves as circumcised were not circumcised on the basis of physical examination [100, 101]. To defend their decision, the author stated “we chose to report the findings of self-reported circumcision. The prevalence of circumcision in Mexico is very low, and the interviewers who did the physical examination may not be accustomed to it and may have been unable to identify its presence.” [100]

This inability of researchers in Mexico to accurately identify the circumcision on physical examination may call into question other studies from Mexico. For example, the study by Vaccarella of Mexican men undergoing vasectomy reported a circumcision rate of 31.7% and was identified as an outlier [86]. This circumcision rate appears to be exaggerated in a country in which circumcision is rare. The studies by Giuliano et al. also recruited a third of their participants from Mexico [102].

Perhaps most concerning is the results reported from the group of researchers from Johns Hopkins, who have after publication of their studies become vociferous advocates of the benefits of circumcision [5, 6]. At the beginning of their randomized clinical trial of circumcision of adult male “volunteers” in Rakai, Uganda, “two subpreputial and shaft swabs were also obtained for future testing of human papillomavirus infection.” [103] In 2011, Tobian et al. reported the results of the HPV cultures of the glans and penile shaft at the 12-month visit of participants in their randomized clinical trial [104]. So, it is not clear why, in 2009, Tobian et al. reported the results of the difference in HPV infections incidence using only samples obtained from the preputial cavity of intact men and the coronal sulcus of circumcised men [82]. Why would Tobian and the research group from Johns Hopkins collect samples from the penile shaft and glans but only report the results from the glans?

Their randomized clinical trial ended in December 2006. In 2004, Weaver et al. published a study that demonstrated the clear differential between intact and circumcised males regarding the likelihood of HPV detection based on sampling the shaft or the glans of the penis [31]. There are only two reasons for the Johns Hopkins researchers to withhold the evidence they collected; either they were not current on the medical literature as it applied the research they were conducting and reporting or they purposely withheld results of the swabs taken from the penile shaft. Neither of the options, incompetence or willful academic misconduct, is appealing. Basically, when Tobian et al. and Auvert et al. reported only on sampling from the glans, they guaranteed a positive finding because the location of HPV on the penis differs according to circumcision status [82, 105].

Research published in December 2008 had demonstrated the HPV viral load varied significantly by anatomic site with the penile shaft having the highest viral loads and being the preferred site for HPV-16 (the most prevalent oncogenic HPV type) replication [106]. There is also the question of whether the glans of the circumcision is too dry to allow accurate sampling [107].

The pertinent question as it relates to a systematic review of the medical literature and meta-analysis is whether studies that report only on cultures taken from the glans of the penis should be included in an analysis and adjusted for or be completely dismissed as invalid?

A couple for studies have indicated that the clearance of HPV takes longer from the intact penis [35, 55, 83, 84]. If this is true, it is unclear what the clinical impact would be. HPV infections on the genitals are transitory. Consequently, if the clearance of the virus takes longer, it would be more likely to be detected in intact men. If sampling is infrequent, prolonged time to viral clearance would result in an overestimate of the incidence of infection as infections of shorter duration could have come and gone and not been detected between scheduled samplings. This is an area that warrants further research.

Finally, the data from the randomized clinical trial of adult male circumcision in Kismu, Kenya, were published in 2012. While swabs were taken from the penile shaft and the glans and the data on circumcision status were collected, the authors failed to report the overall rates of HPV infection by circumcision status [77]. If one back calculates using the rates of infections by the type of penile lesion and rates of the types of lesions by circumcision status and assumes there is no interaction between these factors, there is no statistically significant difference between HPV infection rates based on circumcision status. I wrote a letter to the editor asking that the authors provide the results of the incidence of HPV infection by circumcision status, but the editor refused to publish my letter.

### 4.12. Any Sexually Transmitted Infections

This is the first systematic review of the medical literature looking at the incidence and the prevalence of any STI as opposed to not acquiring an STI based on circumcision status. This analysis indicates that prevalence of acquiring any STI is lower in intact men. Three of the four studies of incidence are consistent with the prevalence date, while one study from New Zealand indicated a significant protective effect. Overall, the incidence data indicate a trend that intact men have a lower incidence of any STI.

When looking at the funnel graph for any STI, the study by Langeni [85] is a clear outlier (Figure 16). When the Langeni study are excluded, the summary odds ratio drops from 0.86 (95% CI = 0.74–1.01) to 0.82 (95% CI = 0.74–0.92). While the odds ratio does not change drastically, the confidence interval is tightened by the 203.41 drop in the chi-square value for between-study heterogeneity. With Langeni included, four of the six measure of publication bias were positive. Once Langeni was excluded, one of the measures of publication bias was positive. Consequently, the analyses of any sexually transmitted disease were performed with Langeni included and with Langeni excluded.

Langeni may also be justifiably excluded because the study reported participant self-report of either GUD or GDS, which might exclude several types of STIs and relied on self-diagnosis in Botswana.

With Langeni excluded, the prevalence of any STI is significantly lower in intact men. When only high-risk populations are considered, the trend is in the same direction, but the difference is not statistically significant. The funnel graph, with the exclusion of Langeni, is fairly symmetric. “Trim and fill” analysis found that no studies needed to be added whether or not Langeni was included.

STIs with genital discharges are more common than genital ulcers, which may explain why the prevalence of any STI is lower in intact males. The ratio of the two general types of STIs within a community may also influence the impact of circumcision on overall risk of having any STI. Differences in these ratios in different populations may also contribute to the between-study heterogeneity.

Identifying and quantitating “any STI” may be problematic as the outcome of interest varied between studies. In some studies, collected data were the recollection of any STI in one's lifetime, while, in others, it was the recollection of any STI within the past 12 months. The range of infections tested for or queried about also varied between studies. Likewise, determinations needed to be made regarding what was an STI. Is a yeast infection a sexually transmitted infection or the result of an imbalance of normal flora? In this analysis, candidal infections as well as infections with *T. vaginalis*, mycoplasma, and ureaplasma were not included. How much this variation affected the summary effect is unknown.

It is clear that despite these methodological concerns that the impact of circumcision on the overall risk of contracting any STI is to increase the overall risk of infection. Because of the hodgepodge of data included in this analysis and disparate results on the incidence of infection, more studies specifically designed to answer this question are needed.

### 4.13. General Findings

Several consistencies in the analyses deserve comment. All of the prevalence analyses showed significant between-study heterogeneity. This reflects the variety of populations, settings, diagnostic methods, and ways of determining circumcision status. Some would argue that given this degree of between-study heterogeneity, any meta-analysis that follows is not worthy of publication. Because of the between-study heterogeneity, one cannot sufficiently emphasize a disclaimer of *caveat emptor*. I have erred on the side that information is good, especially when properly presented. Looking at the data from different perspectives and applying different techniques that might help identify the sources of between-study heterogeneity should guide the reader in how to interpret this information.

The summary effect for the prevalence of every disease was greater in studies of high-risk populations than in studies general populations. This consistent finding, which was often statistically significant, has public policy implications. Calls for population-wide implementation of male circumcision on the grounds that it prevents STIs are not supported by the findings of these analyses. These analyses indicate that if male circumcision has any role (which these analyses also dispute) in reducing the incidence and prevalence of STIs, it should be implemented in easily identifiable high-risk populations. A major problem with infant circumcision is the lack of an accurate method of identifying which infants will find themselves in high-risk population when they become sexually active. Similarly, meta-regression analysis of the studies of HIV incidence and prevalence has found that there is no significant association in general populations but only in high-risk populations [108].

In several analyses, the summary effect of the prevalence of a disease was significantly and positively associated with circumcision prevalence in the population studied. A similar finding has been identified in studies of HIV incidence and prevalence [108]. These findings are consistent with how sexual networks impact the spread STIs [109]. Sexual partners are not found randomly but usually within one's cultural or ethnic group. Since circumcision status has a strong association with religious, tribal, and cultural factors, men with a particular circumcision status will likely have sexual partners from within a group that has a predominance of men with the same circumcision status. The smaller the group, the more quickly the rise and the higher the peak prevalence for a particular STI [109]. Consequently, when circumcision rates are high, intact men would be more likely to be in a smaller ethnic, religious, or cultural group and thus have a higher peak prevalence of a disease. As the circumcision prevalence drops, circumcised men would find themselves in the smaller groups that would be more likely to have a higher peak prevalence of infections.

The lack of a significant association between high-risk HPV infections and circumcision status undermines the argument made by the few who believe that circumcision reduces cancer risk [8, 11, 110]. The lack of an association between HPV, HSV, and other STIs also undermines the analysis published by the same researchers at Johns Hopkins that selectively reported their HPV findings in Africa. They concluded that infant circumcision would save billions of dollars in public health expenditures, but these researchers relied almost exclusively on their own flawed data, which they failed to adjust for lead-time bias or sampling bias [10]. If circumcision increases the overall incidence and prevalence of STIs, how will it save money?

The results of these analyses also further undermine the argument of how the increased risk of HIV infection in intact men is biologically plausible. The plausibility argument is based on several assumptions, all of which are purely speculative. The first is that the inner mucosa of the foreskin is thinner and more prone to abrasions. The second is that the subpreputial space is a breeding ground for sexually transmitted viruses. The third is that the Langerhans cells on the mucosal surface act like HIV-virus magnets pulling the virus into the body [111]. The preputial mucosa is not thinner [112, 113], and circumcised men have a trend toward more penile abrasions (presumably from lack of adequate lubrication) [114]. Langerhans cells are quite efficient in killing HIV cells, which explains the low rate of transmission through sexual contact (approximately 1 in 1000 unprotected acts of coitus) and require activated T cells [115, 116]. Langerhans cells are the first line of mucosal defense. Their presence in the mucosal portion of the prepuce may explain why the overall incidence and prevalence of STIs is lower in intact men. Finally, there is no difference in the incidence and prevalence of HSV or HPV based on circumcision status. The claim that the subpreputial space is a preferential breeding ground for these viruses is also contradicted by the research that found the highest viral replication rates and viral load of HPV on the penile skin [106]. Men with genital ulcers are at greater risk because of the disruption in epithelial integrity at the site of the ulcer and the activation of T cells by the inflammation accompanying the ulcer.

### 4.14. Missed Studies of Interest

There are several studies that reported results that could not be incorporated into the analyses. For example, Urassa et al. reported that they did not find a significant difference in GDS or GUD prevalence in males based on circumcision status but gave no further details [79]. In 1949, Hand reported, without providing his data, no difference in the rate of HSV in soldiers on the basis of circumcision status [25]. A study of 537 sailors examined for gonorrhea before and after shore leave in the Far East found that circumcision status did not significantly affect the susceptibility to gonorrhea but provided no specifics [78].

Because circumcision status based on country of origin is inexact, a Dutch study was excluded that found that men born in the Netherlands, where circumcision is an uncommon practice, had lower rates of STIs than men who immigrated from Turkey, where circumcision is nearly uniformly practiced (one or more STI: OR = 0.30 and 95% CI = 0.12–0.72; HSV: exact OR = 0.37and 95% CI = 0.007 infinity; early syphilis: exact OR = 0.20 and 95% CI = 0.06–0.63; gonorrhea: OR = 0.20 and 95% CI = 0.06–0.63; chlamydia: OR = 0.42 and 95% CI = 0.14–1.37) [117]. These results also support the theory that minority groups have a higher peak prevalence of STIs.

Of historical interest, a study of the cause of deaths in New York City in 1931 found that death from syphilis and related diagnoses was lower in Jews than non-Jews (Poisson regression RR = 0.66 and 95% CI = 0.51–0.86). When only males are considered, the results are similar (Poisson regression RR = 0.66 and 95% CI = 0.49–0.88). If circumcision was a contributing factor, beyond that seen for ethnicity alone, one would expect a significant interaction between ethnicity and gender in which Jewish men would have a lower rate of syphilis than Jewish women. Such an interaction could not be demonstrated (*P* = .6500) [118]. Likewise, Jewish men and women were found less likely to have syphilis in 1882–1883, but, once again, the lack of interaction between ethnicity and gender (*P* = .9007) fails to support circumcision as a contributing factor [119]. The differences in the rates of lues between ethnic groups can be explained by a lack of sexual mixing between the two populations. For example, Christian prostitutes were banned from consorting with Jews [120].

### 4.15. Methodological Choices

This paper did not review the literature for HIV infections for two reasons. First, such a review would be lengthy and best left to another article. Second, most of the study of HIV and circumcision status has taken place in Africa. In that setting that is estimated 20% or more of infections are not spread through sexual contact [121–128]. Using the data from the three African randomized clinical trials in adult males that looked for an association between circumcision and incidence of HIV infection [103, 129, 130], it appears that approximately half of the infections documented in these studies were transmitted through nonsexual means [131]. None of these trials made any attempt to determine the source of HIV infection documented in the trials. Consequently, since it is not clear whether the HIV infections identified in African studies were sexually transmitted or iatrogenic infections, HIV infections were excluded from this paper.

A drawback seen in some observational studies is having a small number of patients with a specific outcome. When this occurs, the parametric assumptions that allow one to make accurate inferences may no longer be valid, resulting inaccurate estimates for odds ratios and 95% confidence intervals. Since these inaccurate calculations of odds ratios and variance can bias summary effects and estimates of variance, including studies with small cell populations can result in inconsistent summary estimates depending on the calculation method used [16]. To minimize any bias introduced by studies with cells with small populations, the odds ratios and confidence intervals were calculated using exact methods.

Some adjustments in the composition of control groups were necessary to provide consistency of methodology between studies. For example, Wilson compared seasoned soldiers to new recruits [23], while Hand's control were men without any exposure to STIs.

In Mallon et al., British men referred to a dermatology specialist for penile problems were compared to a control group of patients without penile problems cared for by the same dermatologists [24]. This is a classic case of referral bias. If primary care providers are less comfortable identifying and treating problems with the complete penis, these men would be overrepresented in a referral dermatology practice. More difficult to explain is the high circumcision rate in the control group: 47.8%. Of the men with penile problems, only 23.0% were circumcised. Yet, in a representative population survey of British adults from the early 1990s, 21.9% of adult males reported being circumcised, with the highest circumcision rate (32.2%) being reported in men aged from 45 to 59 years [132]. In a 2000 British survey, 15.8% of British men reported being circumcised [133]. Clearly, a control group in which 47.8% are circumcised was not representative of the general population.

Using a control group of men without any STI is problematic. First, men without a detectable STI differ in several ways from men who have an STI and introduce a “Berksonian bias” [134]. Some have the mistaken belief that contracting a different STI introduces unidirectional bias [135]. The opposite is likely the case. Excluding men with a different STI is more likely to introduce bias. For example, if, while investigating for association between the prevalence of gonorrhea and circumcision status, all men with syphilis, whether or not they have gonorrhea, are excluded, the measure of association will be biased because intact men presumably have a higher prevalence of syphilis. By excluding a disproportionate number of intact men, the odds ratio for intact men having gonorrhea, after excluding those with syphilis, will be higher than if these men had been included. Similarly, if men with genital warts, which is more common among circumcised men, are excluded, then the odds ratio for intact men having gonorrhea will decrease. In order to justify excluding these men from the analysis, these other conditions would need to be shown to be confounding factors or effect modifiers for gonorrhea. This has not been demonstrated for the diseases in these analyses.

Second, using a disease-free control group discards data collected on men who had an STI other than the infection of interest. Those who participate in medical research allow their medical information to be used and their privacy to be violated. Violating a subject's privacy to collect data and then not use the information excludes useful information and is ethically suspect. Every participant's information should contribute to a study, and so serious deliberation needs to be undertaken before this information is arbitrarily excluded from analysis. If the aim of a study is to consider a specific infection, the data on all patients meeting the inclusion criteria should be incorporated into the analysis. For example, in a cross-sectional study, the characteristics of men with the disease of interest would be compared to the characteristics of men without the illness, regardless if they happen to have a different type of infection.

Finally, it provides a method of comparison that is consistent with the other studies included in the meta-analysis.

Many prefer to use individual patient data in meta-analyses for a variety of reasons [136]. First, not all studies adjust their results for confounding factors. In fact, most studies identified in this paper did not. Second, studies that provided adjusted odds ratios do not consistently adjust for the same factors, so adjusted results from different studies are not comparable. Third, most studies that report adjusted results rarely perform evaluations for collinearity, which can destabilize multivariate models. Circumcision status has been noted in several studies to be a differential factor in the number of lifetime sexual partners, marriage rates, contact with prostitutes, and tobacco and alcohol consumption [137, 138]. If a study were to adjust for one of these factors, they might find that particular factor is significant, circumcision is significant, or both are significant, when the truth is that circumcision is linked to the other factor and the two variables in a multivariate model are describing the same thing. Fourth, when adjusted odds ratios are calculated, the uncertainty (variance) of the estimate increases. When calculating a summary effect, the weight assigned to data from an individual study is the inverse of the variance. An adjusted odds ratio will have a larger variance and give the study less weight when determining the summary effect than the unadjusted odds ratio would. For example, in the study by Laumann et al. [89], the weight assigned to the raw data is from 3.6 to 6.6 times greater than the weight assigned to the adjusted odds ratios. Similarly, in a study by Urassa et al., going from raw data to an adjusted odds ratio increased the variance from 0.000685 to 0.0153 [79]. Subsequently, a much smaller and less rigorous study that reported only raw data would have more impact on the summary effect than a large nationally representative probability sample using adjusted odds ratios. Fifth, adjusted odds ratios are open to manipulation using multivariate logistic regression. Consequently, using raw data will diminish the impact of researcher bias and avoid overfitting the data with multivariate analysis.

One of the most important tasks in performing the literature review is looking for forms of bias and making adjustments to minimize the impact of differential bias. Bias happens, and it is hard to identify and control. Most forms of bias are insidious and difficult to measure. Circumcision status, which is linked to socioeconomic status, may impact healthcare seeking behaviors. If, for example, circumcised men are more likely to visit an STD clinic for reassurance purposes, they would be more likely to be placed in a no disease only control group thus increasing the odds ratio for those intact men and the illness of interest [139].

Lead-time bias was present in all of the data coming from the randomized clinical trials of adult male circumcision in Africa. Because men randomized to immediate circumcision were not exposed to STIs for four to six weeks following their procedures, their exposure to disease was not the same as men who were assigned to later circumcision. While a six-week adjustment to trials scheduled to last from 21 to 24 months wound not appear to be substantial, when the reduced exposure time is accounted for, several of the associations found that these trials were no longer statistically significant. If these findings were robust, adjusting for lead-time bias should not have influenced the interpretation of the results.

What is more concerning is that potential for lead-time bias was overlooked in the planning, funding, analysis, and reporting phases of these projects. The potential for lead-time bias in any cohort study or clinical trial is taught and emphasized in the most basic classes on research design. How was this potential source of bias missed by the highly regarded researchers at Johns Hopkins, the reviewers who approved funding for these studies at the National Institutes of Health, and the editors and peer-reviewers at highly regarded medicals journals such as *The Lancet* and *The New England Journal of Medicine*? To compensate for the deficiencies of these individuals, a *post hoc* adjustment of six weeks lead time was made. Six weeks were chosen to be on the conservative side.

The need to adjust for sampling bias in the studies of HPV is quite apparent. Multiple studies have found that the location of HPV on the penis is differentiated by circumcision status [29–35] and meta-regression has found that studies that sample only the glans have a significant difference in the odds ratio. The problem is that the entire treatment effect reported in studies that sampled only the glans [82, 105] can be attributed to sampling bias [26, 28]. Unfortunately, these studies are widely cited. While it could be argued that failure to sample the penile shaft is a fatal flaw, an adjustment for the number of infections missed is a straight forward solution. Doing so for the studies of disease incidence brought these studies in line with other studies that adequately sampled the genitals. 

Nondifferential misclassification is a concern as the correlation between circumcision status based on patient report and physical examination can vary widely depending on the population studied. [79, 100, 140–145]. Method of determining circumcision status was a significant factor in the meta-regression of studies of the prevalence of HPV. For some study designs, ascertaining circumcision status is not practical. For example, a number of studies of using representative samples of the general population relied on the subject report for circumcision status (Table 1). 

Reliance on the patient report to document an STI introduces a potential for recall bias and may underestimate the incidence of STIs. This would only introduce bias if a differential ability to recall and report medically diagnosed sexually transmitted disease was linked to circumcision status [146]. There is no reason to believe it is.

Searching for sources of bias also occurs in a meta-analysis, particularly for those involving observational studies, when looking at the impact of various factors on between-study heterogeneity. Some consider accounting for contributions to between-study heterogeneity is an obligation for the investigator and the most important task in performing a meta-analysis [37]. It is particularly important for observational studies, which, compared to randomized clinical and controlled trials, are, on average, likely to overestimate the true odds ratio by 30% [147]. Other methods that look to reduce between-study heterogeneity include the search for and the exclusion of studies that contain appreciable outlier data [148], sensitivity analysis, and meta-regression.

Most of the between-study heterogeneity can likely be attributed to methodological limitations in the source studies and the inherent biases in study design. Many of the studies included in these analyses reported information collected at STD clinics. While these clinics provided concentrated clinical material at one location, their clientele does not reflect the characteristics and risk factors for disease seen in the general population and may introduce a selection bias that unduly influences the results generated [109]. Intact and circumcised men may not use these health facilities with equal frequency for similar indications. For example, in the United States and England, men with higher socioeconomic status are more likely to be circumcised and more likely to have an STI treated by a physician in private practice rather than at an STD clinic. Health-seeking behaviors may be different in circumcised men who might be more likely to seek care for minor abrasions thus being placed in control group more frequently than their intact cohorts [134].

### 4.16. Shortcomings of Meta-Analysis

Meta-analysis is an inexact tool and best applied to randomized controlled trials. It has inherent weaknesses when applied to observational studies, so guidelines on how to undertake this process have been published proposed [22]. The validity of a meta-analysis of observational studies is related to study quality. The simple inclusion criteria allowed several studies of less than optimal quality to be included; however, more exclusive criteria can be subject to researcher bias and be manipulated to obtain specific results [15]. The simple inclusion criteria may contribute to the between-study heterogeneity.

The analyses presented in this paper used a random-effects model to determine summary effects and confidence intervals. The alternative, fixed-effects models assume a single true effect common to all studies. Any variation would be attributed only to sampling error. Random-effects models allow for a true random component as a source of variation in effect size between studies as well as sampling error [149]. If between-study heterogeneity is low, the random-effects model will give an estimate and confidence intervals similar to a fixed-effects model. In general, random-effects models are preferred because the assumptions for a fixed-effects model to be accurate are rarely satisfied [150].

One limitation of this systematic review, or any systematic review, is the inability to find all sources of data using any search strategy. All search strategies have an ascertainment bias: the goal is to diminish this bias by finding as many relevant studies as feasible. So, there may be published and unpublished studies that were not included.

The measures of publication bias are a mathematical attempt to quantify the gestalt of looking a funnel graph and determining if it looks like an inverted funnel. Each measure of publication bias has its strengths and weakness [40], but since there are no comparative analyses of the different methods of identifying publication bias, and the gold standard is our gestalt, all of the measures of publication bias should be used [151]. They are often less than helpful. In the analyses published here, the results between the six different measures were often inconsistent, and funnel graphs that looked asymmetric in several instances did not have positive measures of publication bias and did not generate an intervention using “trim and fill” analysis. The trim portion of the “trim and fill” method is handicapped by being based solely on rank, without consideration of study size. Consequently, adjustments for publication bias should be viewed with caution as asymmetry of the funnel plot may be due to factors other than publication bias, and, likewise, results generated to correct for the asymmetry may not reflect a correction for publication bias [151].

## 5. Summary

The results of these meta-analysis should be taken with caution. The trials they are based on come from a number of sources with a number of different methodologies. Some studies employed exemplary methodology, while others were published in high-profile medical journals, such as the *New England Journal of Medicine* and *The Lancet*, and contained serious and possibly fatal methodological flaws. Some forms of the differential bias could be identified and adjusted for, but there are likely many forms of bias that cannot be identified. All of the analyses had significant between-study heterogeneity, which undermines the robustness of any of the findings.

Most specific STIs are not impacted significantly by circumcision status. These include chlamydia, gonorrhea, HSV, and HPV. Syphilis showed mixed results with prevalence studies suggesting intact men were at great risk and incidence studies suggesting the opposite. Intact men appear to be greater risk for GUD while at lower risk for GDS, NSU, genital warts, and the overall risk of any STIs. It is also clear that any positive impact of circumcision on STIs is not seen in general populations. Consequently, the prevention of STIs cannot be rationally interpreted as a benefit of circumcision, and a policy of circumcision for the general population to prevent STIs is not supported by the evidence currently available in the medical literature.

## Figures and Tables

**Table 1:** Attributes of studies meeting the inclusion criteria.

Study STI studied	Location	When	Population	Type of study	Circumcision status	Method of diagnosis
Agot [152]; GDS and GUD	Kenya	From October 1999 to May 2000	18–49 y/o sexually active males unaware of HIV status in circumcising and noncircumcising denominations in Luo ethnic community	Cross-sectional	Physical exam	Report

Auvert et al. [153]; GC, CT, and any	Kisumu, Kenya	From June 1997 to March 1998	General population	Cluster design to randomly select households	Physical exam and self-report	Serology (HSV and syphilis). Urine DNA (GC and CT)

Auvert et al. [105]; HPV and GC	Orange Farm, South Africa	2002–2006	Men interested in a free circumcision	Randomized clinical trial	Intention to treat	Urethral swabs (HPV)

Aynaud et al. [33]; HPV	Paris, France	From March 1991 to September 1992	Men whose female partners had genital condylomata or intraepithelial neoplasia	Cross-sectional study	Physical exam	Colposcopy, viral culture, and biopsy

Aynaud et al. [34]; NSU, CT, GC, HPV, and any	Paris, France	Not documented	Heterosexual HIV-negative men whose female partner has HPV	Cross-sectional study	Physical exam	Chlamydia by PCR, cultures (GC), and HPV by biopsy and penoscopy

Bailey [114]; GDS, GC, and syphilis	Mbale, Uganda	From April to May 1997	General population	Single stage cluster sampling cross section	Report	Report

Baldwin [154]; HPV	Tucson, Arizona	From July 2000 to January 2001	High risk men attending a public STD clinic	Cross-sectional	Physical exam	Swab of glans and sulcus (HPV)

Barile [59]; GUD	Japan	Not documented	US military personnel in Japan	Case control	Physical exam	Clinically

Bassett [155]; HSV	Sydney, Australia	From December 1990 to May 1991	STD clinic	Consecutive sample of heterosexual men	Physical exam	HSV2 by serology

Bleeker [156]; HPV	Amsterdam	From April 2002 to November 2002	18–75 years old. Group A with female partner without CIN. Group B female partner with CIN. Non-STD hospital population	Consecutive sample of male partners of	Physical exam	Swab of glans, sulcus, corona, and frenulum (HPV)

Burundi [72]; GDS, GUD, and any	Burundi	2010	General population from 15 to 49 years old	National representative population survey	Patient report	Patient report

Buvé [61]; syphilis and HSV	Kisumu, Kenya; Ndola, Zambia; Cotonou, Benin; Yaoundé, Cameroon	From June 1997 to March 1998	General population from 15 to 49 years old	Cluster design to randomly select households	Physical exam and self-report	Serology (HSV and Lues)

Bwayo [36]; GDS, GUD, and syphilis	25 miles from Nairobi	From June 1989 to February 1992	Truck drivers enrolled at roadside research clinic	Self-selected convenience sample	Not documented	Report (GDS and GUD) and serology (Lues)

Cameron [97]; GUD versus GDS	Nairobi, Kenya	From March 1986 to December 1987. Followup to March 1988	STD clinic and men who got STD from a prostitute	Prospective cohort study	Physical exam	Not documented

Castellsagué [99]; HPV	Brazil, Thailand, Philippines, Spain, Columbia	1985–1993	Husband or stable partner of woman with cervical cancer or a control woman	Seven separate case-control studies	Physical exam in Brazil, Thailand, and Philippines. Report in Spain and Columbia	PCR for HPV from urethra and glans swabs

Cook [30]; NSU, CT, GC, syphilis, HSV, HPV (warts), and any	Seattle, Washington	From January to December 1988	STD clinic	Chart review of heterosexual men	Chart review (14.3% missing)	Urethral swabs, syphilis by serology, warts, and HSV clinically and warts clinically

Dave [133]; NSU, CT, GC, syphilis, HSV, HPV (warts), and any	Great Britain	2000	General population	Large-scale, stratified, probability sample survey	Report	Report

Dickson et al. [157]; HSV and any	Dunedin, New Zealand	1999	Birth cohort from 1972 to 1973	Prospective cohort repeatedly studied from birth	Not documented	Serology (HSV)

Dickson et al. [51]; any	Dunedin, New Zealand	From 2004 to 2005	Birth cohort from 1972 to 1973	Prospective cohort repeatedly studied from birth	Life-time medical records	Life-time medical records

Dickson et al. [158]; HPV	Dunedin, New Zealand	From 2004 to 2005	Birth cohort from 1972 to 1973	Prospective cohort repeatedly studied from birth	Life-time medical records	Serology for 6, 11, 16, and 18

Dinh [71]; HPV (warts)	United States	From 1999 to 2004	Random sample of general population aged from 18 to 59	National survey NHANES	Patient report using visual aids	Patient report

Diseker [159]; CT, GC, syphilis, and any	Baltimore, Denver, Long Beach, San Francisco	From July 1993 to September 1996	STD clinic	Part of RCT, baseline analysis, and cohort analysis	Physical exam	GC by culture, chlamydia by urine PCR, and syphilis by serology

Donovan [160]; NSU, GC, syphilis, HSV, and HPV (warts)	Sydney, Australia	From December 1990 to May 1991	STD clinic	Consecutive sample of heterosexual men	Physical exam	NGU by clinical picture and microscopy, TPHA for syphilis, HSV by cell culture or clinical criteria, and warts clinically

Fergusson [138]; CT, and any	Christchurch, New Zealand	From 1998 and 2002	Birth cohort from 1977	Prospective cohort repeatedly studied from birth	Report and medical records	Patient report

Ferris [161]; any, warts, CT, HSV, GC, and NSU	Australia	2005	16–64 years old	Representative national sample	Patient report	Patient report

Gebremedhin [52, 162]; any	Africa	2003–2007	General population	18 national demographic health surveys	Patient report	Patient report

Giuliano et al. [102]; HPV	Sao Paulo, Brazil; Morelos, Mexico, Central Florida	2005 and 2006	18–70 years old, no previous warts, and no STD or HIV	Prospective cohort study	Physical exam	Glans, sulcus, shaft, and scrotum

Gottlieb [62]; HSV	Baltimore, Denver, Long Beach, San Francisco	From July 1993 to September 1996	STD clinic	Part of RCT cohort analysis	Physical exam	Serology (HSV)

Gray et al. [44, 163], GDS, CT, GC, Lues, HSV, and GUD	Rakai, Uganda	From November 1994 to October 1998	General population	Randomized cluster of general population	Report	Urine PCR (GC and CT), serology (HSV and syphilis), and clinically (GUD)

Gray et al. [53]; GUD	Rakai, Uganda	Completed December 2006	Men 15–45 who wanted a free circumcision	Randomized controlled trial	Intention to treat	GUD on physical examination

Hand [25]; GC, syphilis, and chancroid	US Naval Hospital St. Albans, NY	1945	Sailors	Not documented	Not documented	Not documented

Harbertson [164]; any STI	Rwanda	From October 2008 to November 2010	Active duty soldiers ≥21 years old in Rwanda Defense Forces from 46 military sites	Cross-sectional	Patient report	Patient report

Hart [69]; chancroid	Australia	1970	Soldiers, STD clinic	Cross-sectional	Physical exam	Clinical diagnosis

Hart [165]; CT and GC	South Australia	From 1988 to 1990	STD clinic	Consecutive sample	Not documented	Chlamydia by enzyme immunoassay and GC by smear and culture

Hernandez [35]; HPV	Hawaii	From July 2004 to December 2006	University students ≥18 years old	Convenience sample	Physical exam	HPV swabbed glans, sulcus, shaft, scrotum, and inner foreskin

Hutchinson [1]; GUD versus GDS	Metropolitan Free Hospital, London	Past year's experience	Men with an STD	Not documented	Jew versus Gentile	Clinically

Kapiga [166]; HSV	Moshi, Tanzania	From June to October 2000	Hotel and bar workers	Randomized sample	Physical exam	Serology (HSV)

Klavs [167]; any	Slovenia	1999–2001	Men 18–49 years old	National probability sample	Report	Patient report

Lajous [100]; HPV	Mexico	From July 2000 to July 2003	Healthy military men	Cross-sectional study	Physical exam performed but analysis based on report	HPV DNA

Langeni [85]; any	Botswana	2001	Men 15–64 who had intercourse	National represented sample	Report	GDS or GDS by report in the past 12 months

Laumann [89]; NSU, CT, GC, syphilis, HSV, and any	United States	1992	Men 18–59 years old	National probability sample	Report	Report

Lavreys [168]; GDS, syphilis, HSV, HPV (warts), GUD, and chancroid	Kenya	From March 1993 to June 1997	HIV negative-truck drivers	Prospective cohort study and convenience sample	Physical exam	Chlamydia by serology assay, TPHA & RPR (syphilis), HSV by serology assay, warts clinically, and chancroid by serology assay

Lloyd [57]; GC, Syphilis, and chancroid	Guy's Hospital, London	From January to June 1932	STD clinic	Convenience sample	Physical exam	Clinically and soft chancre clinically

Lu [84]; HPV	Tucscon, Arizona; Tampa, Florida	From September 2003 to December 2005	18–40 year old sexually active males with no previous genital warts or penile cancer or current STD	Prospective cohort study	Physical exam	Glans, shaft, and scrotum

Mallon [24]; HSV and HPV (warts)	Chelsea and Westminster Hospital, London	1994–1997	Patients referred to a dermatology specialty clinic	Retrospective case control	Physical exam	Not documented

Mandal [169]; HPV	United Kingdom		STD clinic and men with no evidence of clinical anogenital warts	Cross-sectional	Not documented	Cytology of swabs from urethra, glans, shaft, and anorectal

Mattson [170]; any	Kisumu, Kenya	2002–2006	18–24 year olds who wanted a free circumcision	Randomized clinical trial	Physical exam	PCR for GC and CT and culture for *T. vaginalis *

Mehta et al. [171]; GC chlamydia	Kisumu, Kenya	2002–2006	Men interested in a free circumcision	Randomized clinical trial	Intention to treat	Urine for GC and chlamydia

Mehta et al. [172]; HSV, Lues, and GUD	Kisumu, Kenya	2002–2006	18–24 year old men who wanted a free circumcision	Randomized clinical trial	Intention to treat	Serology (Lues and HSV) and Clinically identified (GUD)

Mor [173]; syphilis	San Francisco	From January 1996 to December 2005	STD clinic	Consecutive sample	Physical exam	Not documented

Mujugira [174]; HSV	Botswana, Kenya, Rwanda, South Africa, Tanzania, Uganda, Zambia	From November 2004 and April 2007	HIV-negative partners of women who are HIV and HSV positive	Discordant couples	Physical exam	Serology (HSV)

Müller [175]; HPV	Alexandra, Johannesburg, South Africa	From December 2006 to July 2008	18+ sexually active attending HIV testing clinic	Cross-sectional	Physical exam	Glans, sulcus, and shaft

Mwandi [88]; HSV	Kenya	From August to December 2007	General population from 15 to 64 years old	Representative samples of households	Patient report	Serology (HSV)

Nasio [98]; GUD versus GDS	Nairobi	From January to September 1993	STD clinic	Convenience sample	Physical exam	Report

Newell [176]; GDS, syphilis, and GUD	Mwanza Region, Tanzania	From 1990 to 1991	General population	Random cluster sample survey	Report	Report (GDS and GUD) and serology (syphilis)

Ng'ayo [177]; HSV	Kisumu, Kenya	Not documented	Fishermen along Lake Victoria ≥18 years of age	Random cluster sample cross-sectional survey	Not documented	Serology (HSV)

Ng'ayo [178]; HPV	Kisumu, Kenya	Not documented	Fishermen along Lake Victoria ≥18 years of age	Random cluster sample cross-sectional survey	Physical exam	Swab of glans, corona, shaft, scrotum, and perianal

Nielson [179]; HPV	Tucscon, Arizona; Tampa, Florida	2002–2005	18–40 year old sexually active males with no previous genital warts or penile cancer or current STD	Cross-sectional	Physical exam	Swab of glans, sulcus, shaft, scrotum, perianal area, and urethra (optional)

Obasi [63]; HSV	Rural Mwanza Region, Tanzania	May and June 1993	General population	Nested case-control study within an RCT	Not documented	Type specific ELISA for HSV2

Oglivie [180]; HPV	British Columbia, Canada	Not documented	STD clinic never MSM	Cross-sectional study	Physical exam	Glans, foreskin, shaft, and scrotum

Oriel [29]; HPV (warts)	St. Thomas Hospital, London	From October 1967 to January 1979	STD clinic	Consecutive sample	Physical exam	Not documented

Otieno-Nyunya [181]; Lues	Kenya	2007	General population	Nationally representative population-based serosurvey	Find article	Serology

Parker [140]; NSU, CT, GC, syphilis, HSV, HPV (warts), and any	Perth, Australia	From May to September 1981	STD clinic	Consecutive sample	Report and physical exam	Cultures and microscopy (NSU, CT, and GC), serology (syphilis) culture (HSV) and warts clinically

Partridge [182]; HPV	Seattle, Washington	From June 2003 to March 2006 recruitment	University of Washington students 18–20 years old history of vaginal intercourse	Prospective cohort study	Physical exam	HPV swabs of penile shaft, glans, foreskin, scrotum, and urine.

Rakwar [70]; chancroid	Mombasa, Kenya	Beginning of March 1993	Long-distance truckers	Cross-sectional	Physical exam	Serology and culture of genital ulcers

Reynolds [183]; GC, syphilis, and HSV	Pune, India	1993–2000	STD clinic	Prospective cohort study	Physical exam	Positive gram stain of urethral discharge (GC), RPR, or darkfield (syphilis). Serology (HSV)

Richters [184]; GC, HSV, NSU, and any	Australia	From May 2001 to June 2002	Males 16–59	National probability sample	Report	Report

Rodriguez-Diaz [185]; any, GC, syphilis, HSV, warts, and CT	San Juan, Puerto Rico	From October 2009 to December 2011	STD clinic and Males from 16 to 83 years old	Cross-sectional	Report	Report

Rombaldi [186]; HPV	Caxias do Sul, Brazil	From February 2003 to July 2004	Sexual partners of women with CIN	Prospective, prevalence study	Physical exam	Penoscopy and sampling of urethra, glans, preputial mucosa, and shaft

Schneider [187]; HSV and syphilis	Guntur district of Andhra Pradesh, India	From September 2004 to September 2005	15–49 year olds general populations	Random clusters	Report	Serology

Schrek [58]; GC, syphilis and any	Hines Veteran Hospital, Illinois	1931–1944	World War I veterans	Cross-sectional study	Age at circumcision by report	Report

Seed [188]; GDS, syphilis, GUD and any	Rwanda	From March to October 1991	Steady sexual partner of women enrolled in Project San Francisco	Cross-sectional study	Physical exam	Report (GDS and GUD) and RPR (syphilis)

Shin [189]; HPV	Busan, South Korea	From August 29 to September 30, 2002	University students	Cross section	Report	Continuous swab for HPV DNA from scrotum to top of glans

Simonsen [190]; GUD	Nairobi	From March to December 1986	STD clinic and men with an STD from a prostitute	Convenience sample	Physical exam	Chancroid by culture

Smith [191]; NSU and GC	US military post	From January 1983 to September 1984	US Army personnel	Cross section	Physical exam	CDC criteria and culture

Sobngwi-Tambekou [192]; GC, CT and HSV	Orange Farm, South Africa	2005	Males interest in a free circumcision	Randomized clinical trial	Intention to treat	Urine samples for GC and CT and serology (HSV)

Suligoi [64]; HSV	Garoua, northern Cameroon	From December 1997 to January 1998	General medical outpatients without complaints of STD or HIV	Consecutive sample	Report	HSV by ELISA

Svare [193]; HPV	Copenhagen, Denmark	From March to December 1993	STD clinic	Consecutive sample	Report	Penile swab for PCR

Talukdar [194]; GC and syphilis	Kolkata, India	Not documented	Homeless men 18–49 years old	Cluster design among homeless men	Religion	Urine PCR (GC) TPHA (syphilis)

Taylor [195]; NSU, GC, HSV, and any	Whitechapel Clinic, The London Hospital	From June 1970 to August 1973	STD clinic	Consecutive sample with randomly selected controls	Chart review (10.9% missing)	Clinical diagnosis and HSV2 by culture

Telzak [60]; GUD	New York City	1988–1991	STD clinic	Prospective cohort study	Patient report	Dark field, RPR, culture, Tzanck smear, and clinical diagnosis

Thomas [68]; any	United States Military	From February 1997 to June 2001	HIV-positive cases and HIV negative controls	Case control	Medical records/patient report	Patient report

Tobian et al. [82]; HPV, HSV, syphilis, GDS, and GUD	Rakai, Uganda	Ended December 2006	Men 15–45 who wanted a free circumcision	Randomized clinical trial	Physical exam	Swab of glans and sulcus (HPV) and serology (HSV and syphilis)

Todd [145]; syphilis	Mwanza, Tanzania	From 1991 to 1994	General population	Community randomized trial nested case control	Not documented	RPR and VDRL

Tseng [66]; HPV (warts)	Los Angeles, California	From May 1975 to October 1985	Cases of penile cancer and matched controls and mean age 55.9 and 56.2, respectively	Case control	Patient report	Patient report

Tyndall [137]; GDS and GUD	Nairobi	Not documented	STD clinic and men with genital ulcers	Convenience sample	Physical exam	Report

Uganda [73]; syphilis	Uganda	From February to September 2011	Adults from 15 to 59	National representative population-based survey	Patient report	Serology

Urassa [79]; syphilis and any	Mwanza Region, Tanzania	Study 1: 1990-1991. Study 2: 1994-1995. Study 3: 1994. Study 4: 1991–1993. Study 5: 1992.	Study 1: GP*_._ Study 2: GP. Study 3: fishing villages. Study 4: factory workers. Study 5: GP*_._	Study 1: stratified random cluster sample. Study 2: community study. Study 3: cluster sample survey. Study 4: intake data of cohort study. Study 5: baseline round of longitudinal cohort study to assess community level STD control measures impact on HIV.	Study 1, 2, 3, and 5: report. Study 4: physical exam.	RPR for syphilis and patient report for any STI

Vaccarella [86]; HPV	Mexico, 27 public clinics in 14 states	From January 2003 to September 2004	Men attending vasectomy clinic	Consecutive sample	Physical exam in Mexico	Complete swab from scrotum to urethra

VanBuskirk [32]; HPV	Seattle, Washington	From June 2003 to March 2006 recruitment data through April 2010	University of Washington students 18–20 years old history of vaginal intercourse	Prospective cohort study	Physical exam	HPV swabs of penile shaft, glans, foreskin, scrotum, and urine.

Van Den Eeden [67]; HPV (warts)	Seattle, Washington	From April 1987 to September 1991	Men ≥ 18 years with condylomata acuminata and matched controls in 4 HMO clinics	Case control	Patient report	Clinical exam

Van Wagoner [196]; HSV	Birmingham, Alabama	Not documented	STD clinic and self-identified heterosexual men	Cross-sectional	Physical exam	Serology and culture

Vardas [197]; HPV	Not reported	18 countries in Africa, Asia-Pacific, Europe, Latin America, and North America	Heterosexual men from 16 to 24 years with 1 to 5 lifetime female partners	Cross-sectional	Physical exam	Serology for 6, 11, 16, and 18; swab of penis, scrotum, perineum, and perianal (HPV)

Warner [87]; GDS, GUD, and GUD versus GDS	Baltimore, MD	From 1993 to 2000	Heterosexual African American men undergoing HIV testing at STD clinics	Chart review	Physical exam documented in medical record	Clinical exam

Vaz [198]; syphilis	Maputo, Mozambique	From 1990 to 1991	Prisoners	Convenience sample	Patient report	RPR and FTA

Weaver [31]; HPV	Seattle, Washington	Part 1: from August 1 to October 30, 2000 and part 2: May 3, from 2001 to July 9, 2002	Part 1: heterosexual men 18–25 years old attending STD clinic. Part 2: sexually active undergraduate male students 18–25 years old	Consecutive sample	Physical exam	HPV swabs of penile shaft, glans, foreskin, scrotum, and urine.

Weiss [65]; HSV	Kisumu, Kenya; Ndola, Zambia; Cotonou, Benin; Yaondé, Cameroon	From June 1997 to March 1998	General population	Cluster design to randomly select households	Physical exam and self-report	Serology (HSV)

Wilson [23]; NSU, GC, Lues, and HPV (warts)	Canada	Not documented	Canadian Army VD treatment center	Convenience sample	Not documented	Not documented

Wolbarst [56]; GUD versus GDS	New York City	Before 1914 to 1926	Private patients in urology practice	Convenience sample	Jew versus Gentile	Clinically

Xu [199]; HSV	United States	1999–2004	General population	National health survey	Patient report using visual aids	Serology (HSV)

**Table 2:** Meta-analysis of circumcision status of adult males and incidence of sexually transmitted infections using Poisson regression.

Study	Intact infections/patient years	Circumcised infections/patient years	Relative risk	95% confidence interval
GDS				
Tobian; unadjusted	60/2790	53/2740	1.1118	0.7684–1.6086
Tobian; 6 weeks*	60/2790	53/2423.85	0.9835	0.6797–1.4230
Chlamydia				
Diseker	36/346	88/1109	1.3073	0.8872–1.9267
Mehta	101/2091	88/2027.5	1.1128	0.8362–1.4810
Mehta; 6 weeks*	101/2091	1875.43	1.0294	0.7735–1.3700
SOB	32/1541.75	19/1550.5	1.6938	0.9601–2.9880
SOB; 6 weeks*	32/1541.75	19/1448.27	1.5820	0.8968–2.7910
Summary			**1.2638**	**1.0194–1.5669**
Summary; 6 weeks*			**1.1973**	**0.9648–1.4859**
Gonorrhea				
Diseker	36/346	83/1109	1.3903	0.9402–2.0557
Mehta	74/2102	70/2065	1.0385	0.7490–1.4399
Mehta; 6 weeks*	74/2102	70/1912.92	0.9620	0.6938–1.3339
SOB	91/1541.75	89/1550.5	1.0283	0.7677–1.3772
SOB; 6 weeks*	91/1541.75	89/1448.27	0.9605	0.7171–1.2864
Summary			**1.1053**	**0.9116–1.3402**
Summary; 6 weeks*			**1.0448**	**0.8611–1.2677**
GUD				
Mehta	101/1950	51/1912	1.9418	1.3800–2.7191
Mehta; 6 weeks*	101/1950	51/1753.81	1.7812	1.2720–2.4940
Tobian	75/2790	48/2740	1.5349	1.0681–2.2045
Tobian; 6 weeks*	75/2790	48/2581.92	1.4460	1.0065–2.0774
Summary			**1.7444**	**1.3637–2.2313**
Summary; 6 weeks*			**1.6195**	**1.2660–2.0716**
Syphilis				
Diseker	4/347	6/1109	2.1306	0.5560–7.5504
Mehta	6/1976	7/1897.5	0.8230	0.2766–2.4490
Mehta; 6 weeks*	6/1976	7/1741.73	0.7558	0.2539–2.2481
Tobian	45/4286	50/4166	0.8748	0.5848–1.3087
Tobian; 6 weeks*	45/4286	50/3925.65	0.8243	0.5511–1.2334
Summary			**0.9267**	**0.6429–1.3359**
Summary; 6 weeks*			**0.8738**	**0.6059–1.2600**
HSV				
Dickson	19/2235	13/1512	0.9888	0.4883–2.0019
Mehta	100/1628.5	86/1493.5	1.0664	0.7993–1.4226
Mehta; 6 weeks*	100/1628.5	86/1379.73	0.9852	0.7384–1.3145
SOB	35/1003	23/995	1.5095	0.8921–2.5546
SOB; 6 weeks*	35/1003	23/929.39	1.4100	0.8332–2.3862
Tobian	153/2906.5	114/2888.5	1.3338	1.0466–1.6998
Tobian; 6 weeks*	153/2906.5	114/2704.81	1.2489	0.9800–1.5917
Summary			**1.2302**	**1.0381–1.4581**
Summary; 6 weeks*			**1.1506**	**0.9709–1.3636**
HPV				
Auvert	144/1086.25	90/1125.25	1.5132	1.1651–1.9650
Auvert; 6 weeks*	144/1086.25	90/1051.06	1.4134	1.0883–1.8355
Auvert ADJ^†^	217.50/1086.25	193.03/1125.25	1.0657	0.8786–1.2924
Auvert ADJ; 6 weeks^∗†^	217.50/1086.25	193.03/1051.06	0.9953	0.8207–1.2072
Dickson	54/7830	41/5220	0.8780	0.5851–1.3177
Lajous	37/174	8/36	1.0451	0.4867–2.2441
Lu	7/25.4	56/243.3	1.1967	0.5454–2.6256
Partridge	32/2486	132/7840	0.7645	0.5196–1.1249
Tobian	80/574	42/466	1.5464	1.0644–2.2466
Tobian; 6 weeks*	80/574	42/412.23	1.3679	0.9416–1.9873
Tobian ADJ^†^	120.83/574	90.08/466	1.0889	0.8289–1.4306
Tobian ADJ; 6 weeks^∗†^	120.83/574	90.08/412.23	0.9633	0.7333–1.2655
VanBuskirk	45/124	142/412	1.0530	0.7530–1.4724
Summary			**1.1640**	**1.0097–1.3421**
Summary; 6 weeks*			**1.1184**	**0.9696–1.2902**
Summary ADJ^†^			**1.0113**	**0.8941–1.1439**
Summary ADJ; 6 weeks^∗†^			**0.9591**	**0.8475–1.0852**
Any STI				
Dickson	70/2991	47/1296	0.9591	0.6627–1.3879
Diseker	135/356	475/1109	0.8853	0.7313–1.0718
Fergusson	37/2848	7/1232	2.2864	1.0194–5.1289
Mattson	17/265.5	27/235	0.5583	0.3043–1.0244
Summary			**0.9127**	**0.7801–1.0679**

*Adjusted for a 6-week lead time bias.

^†^Adjusted for sampling bias using data from VanBuskirk et al. [32].

**Table 3:** Studies of the association between circumcision status and the prevalence of genital ulcerative disease versus genital discharge syndrome.

Study	Intact +ve/−ve	Circumcised +ve/−ve	Odds ratio	95% confidence interval	Exact odds ratio	Exact 95% confidence interval
Cameron	56/23	94/120	3.11	1.78–2.63	3.0961	1.7301–5.6797
Hutchinson	165/107	11/47	6.59	3.27–13.27	6.5517	3.1747–14.6549
Nasio	58/20	373/207	1.61	0.94–2.75	1.6083	0.9226–2.9055
Warner	492/2316	1836/14352	1.66	1.49–1.85	1.6606	1.4863–1.8531
Wolbarst	330/420	203/547	2.12	1.71–2.63	2.1161	1.6959–2.6444
Random effects summary effect:					2.2368	1.63–2.24

Heterogeneity chi-square (df = 5) was 17.94 (*P* = .0030).

**Table 4:** Studies of the association between circumcision status and the prevalence of genital discharge syndrome.

Study	Intact +ve/−ve	Circumcised +ve/−ve	Odds ratio	95% confidence interval	Exact odds ratio	Exact 95% confidence interval
Agot	207/237	184/210	1.00	0.76–1.31	0.9968	0.7525–1.3206
Bailey	58/118	65/79	0.60	0.38–0.94	0.5984	0.3657–0.9654
Burundi	48/1612	22/864	1.17	0.70–1.95	1.1693	0.6870–2.0486
Bwayo	88/88	376/383	1.02	0.73–1.41	1.0186	0.7238–1.4336
Gray et al. [44]*	156/4443	33/875	0.92	0.64–1.36	0.9310	0.6312–1.4097
Gray et al. [163]*	503/3967	97/728	0.93	0.64–1.36	0.9516	0.7528–1.2123
Lavreys	47/48	297/354	1.17	0.76–1.80	1.1668	0.7404–1.8383
Newell	77/1279	58/588	0.61	0.43–0.87	0.6105	0.4222–0.8866
Seed	236/358	136/107	0.52	0.38–0.70	0.5159	0.3790–0.7095
Tyndall	86/92	311/321	0.96	0.69–1.35	0.9649	0.6818–1.3646
Warner	2316/2849	14352/21054	1.19	1.12–1.26	1.1925	1.1239–1.2653
Random effects summary effect:					0.8902	0.7277–1.0891

Heterogeneity chi-square (df = 9) was 47.36 (*P* < .0001).

*The Rakai data published in 2004 was used in calculating the summary effect odds ratio.

**Table 5:** Studies of the association between circumcision status and the prevalence of nongonococcal urethritis.

Study	Uncircumcised +ve/−ve	Circumcised +ve/−ve	Odds ratio	95% confidence interval	Exact odds ratio	Exact confidence interval
Aynaud	56/106	9/39	2.29	1.04–5.06	2.2811	0.9954–5.7489
Cook	161/379	721/1515	0.89	0.73–1.10	0.8927	0.7225–1.0996
Dave	169/4664	39/943	0.88	0.61–1.25	0.8762	0.6106–1.2843
Donovan	55/60	81/104	1.18	0.74–1.88	1.1763	0.7177–1.9282
Ferris	34/1567	136/2209	0.35	0.24–0.52	0.3525	0.2333–0.5198
Laumann	21/1097	35/1414	0.77	0.45–1.34	0.7735	0.4252–1.3751
Lavreys	15/80	81/570	1.32	0.73–2.40	1.3189	0.6724–2.4469
Parker	138/452	236/493	0.64	0.50–0.82	0.6380	0.4946–0.8211
Richters	150/3367	369/5092	0.61	0.51–0.75	0.6148	0.5025–0.7492
Smith	NA	NA	0.61	0.50–0.73	0.61	0.50–0.73
Taylor	100/207	42/62	0.71	0.45–1.13	0.7137	0.4405–1.1624
Wilson	140/860	45/259	0.94	0.65–1.35	0.9370	0.6449–1.3807
Random effects summary effect:					0.76	0.63–0.92

Heterogeneity chi-square (df = 11) was 39.78 (*P* < .0001).

**Table 6:** Studies of the association between circumcision status and the prevalence of *Chlamydia trachomatis*.

Study	Intact +ve/−ve	Circumcised +ve/−ve	Odds ratio	95% confidence interval	Exact odds ratio	Exact confidence interval
Auvert	9/340	3/132	1.32	0.31–4.37	1.1644	0.2848–6.7884
Aynaud	8/154	1/47	2.44	0.30–20.03	2.4335	0.3125–110.6090
Cook	34/506	147/2089	0.95	0.65–1.40	0.9549	0.6293–1.4145
Dave	72/4761	12/970	1.22	0.66–2.26	1.2224	0.6554–2.4849
Diseker	72/212	240/622	0.88	0.65–1.20	0.8803	0.6382–1.2057
Fergusson	NA	NA	2.50	0.73–8.53	2.50	0.73–8.53
Ferris	30/1571	59/2294	0.74	0.48–1.16	0.7425	0.4596–1.1774
Gray et al. [44]*	71/2131	17/421	0.83	0.48–1.42	0.8252	0.4751–1.5104
Gray et al. [163]*	53/2589	15/462	0.63	0.35–1.13	0.6306	0.3466–1.2152
Hart	251/2725	330/4686	1.31	1.10–1.55	1.3079	1.0979–1.5567
Laumann	0/1118	36/1413	0.02	0.00–0.28	0.0246	0–0.1368
Lavreys	15/33	31/36	0.53	0.24–1.15	0.5308	0.2238–1.2241
Parker	37/553	45/684	1.02	0.65–1.59	1.0170	0.6303–1.6322
Richters	74/3392	116/5218	0.98	0.73–1.32	0.9813	0.7206–1.3295
Rodriguez-Diaz	41/405	20/194	0.98	0.56–1.72	0.9820	0.5452–1.8198
Random effects summary effect:					0.9099	0.72–1.15

Heterogeneity chi-square (df = 12) was 35.53 (*P* = .0007).

*The Rakai data published in 2004 was used in calculating the summary effect odds ratio.

**Table 7:** Studies of the association between circumcision status and the prevalence of gonorrhea.

Study	Intact +ve/−ve	Circumcised +ve/−ve	Odds ratio	95% confidence interval	Exact odds ratio	Exact confidence interval
Aynaud	1/161	0/48	0.90	0.04–22.47	0.2963	0.0076–[11.5518]
Bailey	58/118	55/89	0.80	0.50–1.26	0.7959	0.4892–1.2944
Cook	87/453	175/2061	2.26	1.72–2.98	2.2616	1.6436–3.0031
Dave	53/4780	15/967	0.71	0.40–1.27	0.7148	0.3950–1.3714
Diseker	110/212	294/622	1.09	0.84–1.44	1.0977	0.8299–1.4474
Donovan	8/107	19/166	0.65	0.28–1.55	0.6541	0.2388–1.6331
Ferris	29/1573	52/2302	0.82	0.52–1.29	0.8162	0.4972–1.3164
Gray et al. [44]*	25/2177	3/435	1.67	0.50–5.54	1.6649	0.5046–8.6526
Gray et al. [163]*	29/2613	4/473	1.31	0.46–3.75	1.3123	0.4578–5.1610
Hand (black)	473/250	71/51	1.36	0.92–2.01	1.3585	0.8987–2.0434
Hand (white)	399/388	123/82	0.69	0.50–0.94	0.6858	0.4947–0.9474
Hart	56/2920	48/4968	1.98	1.35–2.93	1.9848	1.3217–2.9904
Laumann; 1–4 partners	9/440	12/542	0.92	0.39–2.21	0.9239	0.3405–2.4141
Laumann; 5–20 partners	64/380	58/480	1.39	0.95–2.04	1.3934	0.9361–2.0775
Laumann; 21+ partners	37/153	55/178	0.78	0.49–1.25	0.7831	0.4743–1.2826
Lavrey	14/81	88/563	1.11	0.60–2.04	1.1056	0.5541–2.0721
Lloyd	203/178	75/43	0.65	0.43–1.00	0.6544	0.4161–1.0203
Parker	54/536	43/686	1.61	1.06–2.44	1.6067	1.0385–2.4983
Reynolds	110/1197	7/184	2.42	1.11–5.27	2.4145	1.1090–6.2430
Richters	85/3471	112/5338	1.17	0.88–1.55	1.1671	0.8669–1.5666
Rodriguez-Diaz	59/387	28/186	1.01	0.63–1.64	1.0127	0.6122–1.7074
Schrek (white)	22/130	10/26	0.44	0.19–1.04	0.4423	0.1750–1.1733
Schrek (black)	50/73	19/26	0.94	0.47–1.87	0.9376	0.4447–2.0000
Smith	NA	NA	1.14	0.92–1.41	1.14	0.92–1.41
Talukdar	19/345	10/92	0.51	0.23–1.13	0.5075	0.2157–1.2662
Taylor	72/235	21/83	1.21	0.70–2.09	1.2104	0.6846–2.2069
Wilson	640/360	229/75	0.58	0.44–0.78	0.5825	0.4291–0.7847
Random effects summary effect:					1.0272	0.86–1.23

Heterogeneity chi-square (df = 25) was 88.81 (*P* < .0001).

*The Rakai data published in 2004 was used in calculating the summary effect odds ratio.

**Table 8:** Studies of the association between circumcision status and the prevalence of genital ulcerative disease.

Study	Intact +ve/−ve	Circumcised +ve/−ve	Odds ratio	95% confidence interval	Exact odds ratios	Exact confidence interval
Agot	133/312	93/301	1.38	1.01–1.88	1.3792	1.0020–1.9032
Barile	32/3	15/32	22.76	6.00–86.29	21.7418	5.5279–128.4283
Burundi	63/1597	17/869	2.02	1.17–3.47	2.0160	1.1564–3.6993
Bwayo	58/118	179/583	1.60	1.12–2.29	1.6000	1.0988–2.3141
Gray et al. [44]*	297/4302	65/843	0.90	0.68–1.18	0.8954	0.6751–1.2021
Gray et al. [163]*	383/4087	64/761	1.11	0.85–1.47	1.1143	0.8428–1.49261
Lavreys	13/82	46/605	2.09	1.08–4.02	2.0825	0.9888–4.1281
Newell	52/1304	18/628	1.39	0.81–2.40	1.3911	0.7926–2.5488
Seed	142/452	41/202	1.55	1.05–2.27	1.5470	1.0407–2.3358
Simonsen	23/47	35/196	2.74	1.48–5.07	2.7297	1.4016–5.2706
Telzak	239/105	211/203	2.19	1.62–2.96	2.1876	1.6056–2.9905
Tyndall	48/130	150/482	1.19	0.81–1.73	1.1862	0.7937–1.7548
Warner	492/4673	1836/33570	1.93	1.73–2.14	1.9250	1.7300–2.1378
Random effects summary effect:					1.6760	1.3926–2.0170

Heterogeneity chi-square (df = 11) was 31.09 using MH (*P* = .0011).

*The Rakai data published in 2004 was used in calculating the summary odds ratio.

**Table 9:** Studies of the association between circumcision status and the prevalence of syphilis.

Study	Intact +ve/−ve	Circumcised +ve/−ve	Odds ratio	95% confidence interval	Exact odds ratio	Exact confidence interval
Bailey	7/169	6/138	0.95	0.31–2.90	0.9528	0.2673–3.5173
Buvé: Kismu	17/393	0/156	13.92	0.83–232.90	9.4870	1.1611–[55.8609]^†^
Buvé: Ndola	57/480	7/48	0.81	0.35–1.88	0.8146	0.3442–2.2355
Bwayo RPR*	14/162	42/729	1.50	0.80–2.81	1.4993	0.7380–2.8812
Bwayo TPHA*	38/75	106/351	1.68	1.07–2.62	1.6761	1.0403–2.6740
Cook	20/520	13/2223	6.69	3.27–13.70	6.5705	3.0874–14.4743
Dave	10/4823	3/979	0.68	0.19–2.46	0.6767	0.1738–3.8332
Diseker	10/212	18/622	1.62	0.74–3.69	1.6290	0.6608–3.7908
Donovan	1/114	2/183	0.80	0.07–8.95	0.8032	0.0135–15.5915
Gray et al. [44]*	482/4117	93/815	1.03	0.81–1.30	1.0260	0.8091–1.3116
Gray et al. [163]*	446/3917	96/709	0.84	0.67–1.06	0.8410	0.6629–1.0750
Hand	420/1090	108/219	0.78	0.60–1.01	0.7815	0.6007–1.0204
Laumann	12/1106	13/1312	1.10	0.50–2.41	1.0950	0.4546–2.6147
Lavreys	11/84	48/603	1.65	0.82–3.29	1.6438	0.7400–3.3705
Lloyd	81/300	25/93	1.00	0.61–1.66	1.0044	0.5937–1.7413
Mor	192/13838	384/36290	1.31	1.10–1.56	1.3112	1.0956–1.5651
Newell	125/1229	45/597	1.35	0.95–1.92	1.3491	0.9379–1.9695
Otieno-Nyunya	NA	NA	2.2	1.3–3.7	2.2	1.3–3.7
Parker	9/581	3/726	3.75	1.01–13.91	3.7452	0.9292–216041
Reynolds	128/1639	9/151	1.31	0.65–2.63	1.3101	0.6510–2.9907
Rodriguez-Diaz	68/378	37/177	0.86	0.56–1.33	0.8608	0.5448–1.3756
Schneider	107/5049	25/986	0.84	0.54–1.30	0.8359	0.5335–1.3562
Schrek white	10/142	1/35	2.46	0.31–19.90	2.4557	0.3293–109.9779
Schrek black	19/104	6/39	1.19	0.44–3.19	1.1863	0.4155–3.9024
Seed	24/570	10/233	0.98	0.46–2.08	0.9811	0.4438–2.3363
Talukdar	25/339	8/94	0.87	0.38–1.98	0.8668	0.3640–2.2979
Todd	187/354	39/137	1.86	1.25–2.76	1.8541	1.2310–2.8387
Uganda	149/6643	37/2452	1.49	1.03–2.14	1.4864	1.0276–2.1990
Urassa	775/3282	155/772	1.18	0.97–1.42	1.1761	0.9707–1.4311
Vaz	74/748	29/433	1.48	0.95–2.31	1.4767	0.9321–2.3934
Wilson	90/910	10/294	2.91	1.49–5.66	2.9059	1.4838–6.3494
Random effects summary effect:					1.3036	1.1103–1.5306

Heterogeneity chi-square (df = 28) was 67.70 (*P* < .0001).

*The RPR data from the Bwayo study and the 2004 Radkai data were used in calculating the summary odds ratios.

^†^Brackets have infinite confidence interval replaced with value equidistant from the odds ratio as the measured confidence interval.

**Table 10:** Studies of the association between circumcision status and the prevalence of genital herpes/herpes simplex virus type 2.

Study	Intact +ve/−ve	Circumcised +ve/−ve	Odds ratio	95% confidence interval	Exact odds ratio	Exact confidence interval
Bassett	69/36	125/70	1.07	0.65–1.34	1.0731	0.6348–1.8283
Buvé	136/199	36/96	1.82	1.17–2.83	1.8202	1.1512–2.9206
Cook	49/491	205/2031	0.99	0.70–1.37	0.9887	0.6977–1.3795
Dave	48/4785	11/971	0.89	0.46–1.71	0.8855	0.4514–1.8979
Dickson	19/241	13/162	0.98	0.47–2.04	0.9825	0.4460–2.2295
Donovan	21/94	27/158	1.31	0.70–2.44	1.3061	0.6618–2.5514
Ferris	28/1594	55/2317	0.74	0.47–1.17	0.7401	0.4499–1.1930
Gottlieb	27/295	74/724	0.90	0.56–1.42	0.8956	0.5424–1.4419
Gray	395/160	76/43	1.40	0.92–2.12	1.3961	0.8962–2.1560
Kapiga	3/8	57/138	1.10	0.28–4.30	0.9083	0.1499–3.9591
Laumann	9/1109	22/1427	0.53	0.24–1.15	0.5265	0.2125–1.1955
Lavreys	20/28	32/33	0.74	0.35–1.56	0.7386	0.3237–1.6679
Mallon	8/267	0/82	5.24	0.30–91.81	3.3651	0.5116–[22.1343]*
Mujugira	669/358	760/483	1.19	1.00–1.41	1.1875	0.9966–1.4156
Mwandi	396/546	1320/4355	2.39	2.07–2.76	2.3925	2.0671–2.7677
Ng'ayo	146/86	14/4	0.49	0.15–1.52	0.4863	0.1129–1.6142
Obasi	25/77	5/25	1.62	0.56–4.69	1.6179	0.5292–5.9872
Parker	60/530	44/685	1.76	1.18–2.64	1.7617	1.1535–2.7079
Reynolds	178/1096	14/111	1.29	0.72–2.30	1.2875	0.7157–2.4867
Richters	68/3476	138/5347	0.76	0.57–1.02	0.7580	0.5566–1.0241
Rodriguez-Diaz	48/398	28/186	0.80	0.49–1.32	0.8014	0.4755–1.3715
Schneider	278/4878	80/931	0.66	0.51–0.86	0.6633	0.5101–0.8702
Suligoi	2/5	18/57	1.27	0.23–7.10	1.2628	0.1112–8.5647
Taylor: UK	102/180	20/59	1.67	0.95–2.93	1.6694	0.9283–3.1007
Taylor: caribbean	50/70	2/12	4.29	0.92–20.00	4.2480	0.8863–40.7423
Taylor: other	36/56	4/33	5.30	1.73–16.24	5.2451	1.6604–22.0974
Van Wagoner ≤ 25 years old	13/31	64/130	0.85	0.42–1.74	0.8524	0.3820–1.8147
Van Wagoner ≥ 26 years old	45/8	77/55	4.02	1.76–9.19	3.9905	1.6878–10.5916
Weiss: Cotonou	1/9	102/751	0.81	0.10–6.52	0.8183	0.0185–6.0119
Weiss: Yaoundé	1/7	235/644	0.39	0.05–3.20	0.3918	0.0087–3.0759
Weiss: Kisumu	161/264	41/117	1.74	1.16–2.61	1.7387	1.1418–2.6833
Weiss: Ndola	194/359	22/32	0.79	0.44–1.39	0.7863	0.4294–1.4627
Xu	146/919	323/2462	1.21	0.98–1.49	1.2109	0.9747–1.4993
Random effects summary effect:					1.1522	0.95–1.40

Heterogeneity chi-square (df = 32) was 152.13 (*P* < .0001).

*Brackets have infinite confidence interval replaced with value equidistant from the odds ratio as the measured confidence interval.

**Table 11:** Studies of the association between circumcision status and the prevalence of chancroid.

Study	Intact +ve/−ve	Circumcised +ve/−ve	Odds ratio	95% confidence interval	Exact odds ratio	Exact confidence interval
Hand; black	60/663	18/104	0.57	0.30–0.92	0.5234	0.2908–0.9807
Hand; white	55/732	5/200	3.01	1.19–7.61	3.0030	1.1890–9.7420
Hart [69]	NA	NA	4.76	3.45–7.14	4.76	3.44–7.14
Lavreys	10/46	91/259	0.62	0.30–1.28	0.6194	0.2674–1.3100
Lloyd	9/372	1/117	2.83	0.35–22.58	2.8263	0.3846–125.1128
Rakwar	NA	NA	0.82	0.50–1.16	0.82	0.50–1.16
Random effects summary effect:					1.33	0.52–1.33

Heterogeneity chi-square (df = 5) was 59.71 (*P* < .0001).

**Table 12:** Studies of the association between circumcision status and the prevalence of genital warts.

Study	Intact +ve/−ve	Circumcised +ve/−ve	Odds ratio	95% confidence interval	Exact odds ratio	Exact confidence interval
Cook	51/489	412/1824	0.46	0.34–0.63	0.4618	0.3326–0.6307
Dave	175/4659	37/945	0.95	0.66–1.37	0.9594	0.6646–1.4173
Dinh	28/1127	133/2822	0.53	0.35–0.80	0.5272	0.3356–0.8026
Donovan	20/95	30/155	1.09	0.58–2.02	1.0874	0.5517–2.1075
Ferris	45/1578	107/2263	0.60	0.42–0.86	0.6032	0.4135–0.8677
Lavreys	3/92	16/635	1.29	0.37–4.53	1.2937	0.2370–4.6463
Mallon	29/246	7/75	1.26	0.53–3.00	1.2623	0.5139–3.5538
Mandal	22/66	6/11	0.61	0.20–1.85	0.6142	0.1815–2.2693
Parker	45/545	52/677	1.07	0.71–1.63	1.0749	0.6931–1.6617
Richters	110/3429	194/5297	0.88	0.69–1.11	0.8759	0.6840–1.1171
Rodriguez-Diaz	54/392	40/174	0.60	0.38–0.94	0.5997	0.3753–0.9640
Tseng: cases	5/56	8/31	0.35	0.35–1.15	0.3499	0.0825–1.3348
Tseng: controls	3/46	3/48	1.04	0.20–5.44	1.0430	0.1328–8.1928
Van Den Eeden	14/72	25/126	0.98	0.48–2.00	0.9801	0.4656–2.1052
Wilson	18/982	0/304	11.47	0.96–190.85	7.8664	1.3505–[45.8203]*
Random effects summary effect:					0.8225	0.65–1.04

*Brackets have infinite confidence interval replaced with value equidistant from the odds ratio as the measured confidence interval.

**Table 13:** Studies of the association between circumcision status and the prevalence of genital human papillomavirus infection.

Study	Intact +ve/−ve	Circumcised +ve/−ve	Odds ratio	95% confidence interval	Exact odds ratio	Exact confidence interval
Aynaud et al. [33]	383/354	119/144	1.31	0.99–1.74	1.3089	0.9773–1.7551
Aynaud et al. [34]	93/69	20/28	1.89	0.98–3.62	1.8812	0.9354–3.8412
Baldwin	46/112	46/186	1.66	1.04–2.66	1.6585	1.0074–2.7333
Bleeker; group A	A: 18/52	3/10	1.15	0.29–4.67	1.1519	0.2562–7.2315
Bleeker; group B	93/67	8/2	0.35	0.07–1.69	0.3489	0.0350–1.8257
Castellsagué; Brazil	40/63	1/5	3.17	0.36–28.18	3.1470	0.3344–153.8345
Castellsagué; Columbia	52/183	0/4	2.57	0.14–48.60	1.4849	0.1824–[12.0884]*
Castellsagué; Philippines	2/20	12/221	1.84	0.38–8.81	1.8362	0.1870–9.1816
Castellsagué; Spain	37/278	1/36	4.79	0.64–35.99	4.7785	0.7551–199.6211
Castellsagué; Thailand	35/ 136	2/35	4.50	1.03–19.64	4.4810	1.0600–40.2844
Giuliano et al. [102]; any HPV^†^	NA	NA	0.97	0.68–1.39	0.97	0.68–1.39
Giuliano et al. [102]; high risk HPV^#^	NA	NA	0.93	0.63–1.33	0.93	0.63–1.33
Hernandez	14/44	52/144	0.88	0.44–1.74	0.8816	0.4113–1.8042
Lajous	365/465	28/67	1.88	1.18–2.98	1.8770	1.1631–3.0978
Mandal	22/66	6/11	0.61	0.20–1.85	0.6142	0.1815–2.2693
Müller; any HPV^†^	125/29	35/19	2.34	1.17–4.66	2.3092	1.0971–4.8997
Müller; high risk^#^	80/74	23/31	1.46	0.78–2.72	1.4545	0.7454–2.8691
Müller; low risk	124/30	34/20	2.43	1.23–4.81	2.4198	1.1521–5.0476
Ng'ayo	136/96	8/10	1.77	0.67–4.65	1.7667	0.6028–5.3567
Nielson; any HPV^†^	38/36	199/190	1.01	0.61–1.66	1.0078	0.5942–1.7117
Nielson; high risk^#^	23/51	112/227	0.91	0.53–1.57	0.9142	0.5062–1.6133
Oglivie; any HPV^†^	89/41	94/38	0.88	0.52–1.49	0.8780	0.4997–1.5397
Oglivie; high risk^#^	25/105	38/94	0.59	0.33–1.05	0.5902	0.3160–1.0877
Oriel	151/69	40/28	1.53	0.87–2.68	1.5295	0.8362–2.7769
Rombaldi	47/42	7/3	0.48	0.12–1.97	0.4830	0.0758–2.2850
Shin	3/40	29/296	0.77	0.22–2.63	0.7660	0.1429–2.6506
Svare	84/89	4/20	4.78	1.57–14.55	4.6869	1.4852–19.6399
Vaccarella	62/470	6/241	5.30	2.26–12.42	5.2905	2.2526–15.1850
Vardas; any HPV	417/1598	247/905	0.96	0.80–1.14	0.9561	0.7981–1.1469
Vardas; high risk	161/1854	115/1037	0.78	0.61–1.01	0.7831	0.6050–1.0161
Weaver STD clinic	0/3	10/17	0.24	0.01–5.08	0.4826	[0.0478]* –4.8700
Weaver University students	17/42	82/176	0.87	0.47–1.62	0.8691	0.4365–1.6708
Random effects summary effects:				Any HPV	1.2411	1.02–1.51
				High-risk HPV	1.1661	0.94–1.45 8
				Selective HPV	1.0128	0.80–1.2

*Brackets indicate that an infinite upper confidence interval was replaced with value equidistant from the odds ratio estimate as the lower confidence interval.

^†^Data used in calculating Any HPV summary effect.

^#^Data used in calculating high risk HPV summary effect.

Any HPV: Heterogeneity chi-square (df = 24) 39.98 (*P* = .0215).

High-risk HPV: Heterogeneity chi-square (df = 24) 45.27 (*P* = .0054).

Selective HPV: Heterogeneity chi-square (df = 15) 28.82 (*P* = .0164).

**Table 14:** Studies of the association between circumcision status and the prevalence of any sexually transmitted infection versus no sexually transmitted infections.

Study	Intact +ve/−ve	Circumcised +ve/−ve	Odds ratio	95% confidence interval	Exact odds ratio	Exact confidence interval
Auvert	82/279	19/122	1.89	1.10–3.25	1.8850	1.0763–3.4396
Aynaud	30/132	7/41	1.33	0.54–3.26	1.3294	0.5208–3.8565
Burundi	101/1559	33/853	1.67	1.12–2.50	1.6743	1.1091–2.5848
Cook	342/198	1449/787	0.94	0.77–1.14	0.9382	0.7685–1.1472
Dave	522/4311	109/873	0.97	0.78–1.21	0.9698	0.7769–1.2187
Diseker	291/212	869/622	0.98	0.80–1.21	0.9825	0.7967–1.2128
Ferris	166/1458	409/1965	0.55	0.45–0.66	0.5471	0.4483–0.6654
Gebremedhin	1178/21926	2623/43363	0.89	0.83–0.95	0.8882	0.8269–0.9536
Harbertson	185/360	371/304	0.42	0.33–0.53	0.4214	0.3311–0.5353
Klavs	38/682	2/35	0.98	0.23–4.21	0.09751	0.2348–8.6761
Langeni	6931/173523	927/35099	1.51	1.41–1.62	1.5124	1.4106–1.6229
Laumann 1–4 partners	15/434	26/528	0.70	0.37–1.34	0.7021	0.3411–1.3960
5–20 partners	80/364	102/436	0.94	0.68–1.30	0.9395	0.6696–1.3152
21+ partners	61/129	95/138	0.69	0.46–1.03	0.6875	0.4502–1.0457
Parker	350/240	404/325	1.17	0.94–1.46	1.1730	0.9360–1.4707
Richters	487/3460	929/4736	0.72	0.12–0.81	0.7176	0.6362–0.8086
Rodriguez-Diaz	293/153	157/57	0.70	0.48–1.00	0.6956	0.4752–1.0104
Schrek white	26/126	10/26	0.54	0.23–1.25	0.5385	0.2176–1.4070
Schrek black	58/65	22/23	0.93	0.47–1.85	0.9333	0.4451–1.9595
Seed	378/216	177/66	0.65	0.47–0.91	0.6529	0.4620–0.9159
Taylor	251/56	87/17	0.88	0.48–1.59	0.8761	0.4521–1.6293
Thomas			1.08	0.52–2.26	1.08	0.52–2.26
Urassa 1	117/1239	70/572	0.77	0.56–1.05	0.7717	0.5592–1.0711
Urassa 2	291/1854	84/374	0.70	0.54–0.91	0.6989	0.5322–0.9246
Urassa 3	85/262	58/119	0.67	0.45–0.99	0.6662	0.4392–1.0133
Urassa 4	29/799	23/723	1.14	0.65–1.99	1.1408	0.6308–2.0853
Urassa 5	355/4409	101/987	0.79	0.62–0.99	0.7869	0.6220–1.0023
Random effects summary effect:					0.8627	0.7368–1.0102
—without Langeni					0.8248	0.7358–0.9245

Heterogeneity chi-square (df = 26) was 303.00 (*P* < .0001); without Langeni chi-square (df = 25) was 99.59 (*P* < .0001).

**Table 15:** Impact of removing outlying studies on between-study heterogeneity and summary effects.

Outlier studies	Chi-square (*P* value)	Adjusted odds ratio (95% confidence interval)	Heterogeneity chi-square (df, *P* value)
GUD versus GDS		**2.24 (1.63–2.24)**	**17.20 (4, .0068)**
Warner	7.85 (.0051)	2.65 (1.67–4.18)	9.35 (3, .0250)
Hutchinson	10.08 (.0015)	1.92 (1.53–2.40)	7.12 (3, .0681)
Hutchinson and Warner	14.87 (df = 2) (.0006)	2.15 (1.68–2.74)	2.33 (2, .3121)
GDS		**0.8902 (0.73–1.09)**	**47.36 (9, <.0001)**
Bailey	6.223 (.0125)	0.9209 (0.75–1.13)	41.13 (8, <.0001)
Newell	9.94 (.0016)	0.9279 (0.76–1.13)	37.42 (8, <.0001)
Seed	23.54 (<.0001)	0.9614 (0.81–1.13)	23.82 (8, .0025)
Warner	26.08 (<.0001)	0.8482 (0.70–1.03)	21.28 (8, .0065)
Warner and Seed	37.30 (df = 2) (<.0001)	0.91.47 (0.79–1.07)	10.06 (7, .1850)
NSU		**0.76 (0.63–0.92)**	**39.78 (11, <.0001)**
Lavreys	4.02 (.0450)	0.74 (0.61–0.89)	35.76 (10, <.0001)
Donovan	4.24 (.0394)	0.74 (0.61–0.92)	35.54 (10, .0001)
Cook	5.94 (.0148)	0.75 (0.61–0.92)	33.84 (10, .0002)
Aynaud	6.25 (.0124)	0.73 (0.61–0.88)	33.53 (10, .0002)
Ferris	12.92 (.0003)	0.80 (0.68–0.95)	26.86 (10, .0027)
Ferris and Aynaud	18.78 (df = 2) (<.0001)	0.77 (0.66–0.91)	21.00 (9, .0126)
Chlamydia		**0.9099 (0.72–1.15)**	**35.53 (13, .0007)**
Hart [165]	10.83 (.0010)	0.8605 (0.67–1.10)	24.70 (12, .0163)
Laumann	18.43 (<.0001)	0.9920 (0.85–1.16)	17.10 (12, .1460)
Laumann and Hart	27.78 (df = 2) (<.0001)	0.9362 (0.87–1.00)	7.75 (11, .7357)
Gonorrhea		**1.03 (0.86–1.23)**	**88.81 (25, <.0001)**
Lloyd	5.23 (.0222)	1.05 (0.88–1.26)	83.58 (24, <.0001)
Hand	9.16 (df = 2) (.0102)	1.04 (0.86–1.25)	79.55 (23, <.0001)
Hart [165]	9.42 (.0021)	0.99 (0.83–1.19)	79.39 (24, <.0001)
Wilson	18.26 (<.0001)	1.07 (0.90–1.26)	70.55 (24, <.0001)
Cook	30.10 (<.0001)	0.99 (0.84–1.15)	58.71 (24, .0002)
Cook and Wilson	44.32 (df = 2) (<.0001)	1.03 (0.89–1.19)	44.49 (23, .0669)
GUD		**1.6760 (1.39–2.02)**	**31.09 (11, .0011)**
Tyndall	4.21 (.0402)	1.7326 (1.43–2.10)	26.88 (10, .0027)
Warner	6.48 (.0109)	1.6492 (1.33–2.05)	24.61 (10, .0109)
Barile	7.64 (.0057)	1.6308 (1.38–1.92)	23.45 (10, .0057)
Gray	10.33 (.0013)	1.7581 (1.48–2.09)	20.76 (10, .0228)
Gray and Warner	12.61 (df = 2) (.0018)	1.7385 (1.40–2.17)	18.48 (9, .0300)
Gray and Barile	17.73 (df = 2) (.0001)	1.7334 (1.51–1.99)	13.36 (9, .1470)
Syphilis		**1.3036 (1.11–1.53)**	**67.70 (28, <.0001)**
Todd	4.18 (.0409)	1.2766 (1.08–1.50)	63.52 (27, <.0001)
Wilson	4.98 (.0256)	1.2704 (1.08–1.49)	62.72 (27, .0001)
Buvé; Kismu	5.24 (.0221)	1.2804 (1.10–1.50)	62.46 (27, .0001)
Otieno	5.37 (.0204)	1.2685 (1.08–1.49)	62.33 (27, .0001)
Gray	9.05 (.0026)	1.3439 (1.14–1.58)	58.65 (27, .0004)
Hand	10.95 (.0009)	1.3467 (1.15–1.58)	56.75 (27, .0007)
Cook	17.52 (<.0001)	1.2304 (1.07–1.42)	50.18 (27, .0043)
Cook and Hand	27.55 (df = 2) (<.0001)	1.2704 (1.11–1.45)	40.15 (26, .0377)
Chancroid		**1.3321 (0.52–1.33)**	**59.71 (5, <.0001)**
Lloyd	4.83 (.0279)	1.5961 (0.5418–4.7022)	54.88 (4, <.0001)
Hand	11.69 (df = 2) (.0029)	1.4490 (0.4186–5.0152)	48.02 (3, <.0001)
Rakwar	14.74 (.0001)	1.5289 (0.4499–5.1956)	44.97 (4, <.0001)
Hart [69]	52.23 (<.0001)	0.8177 (0.5092–1.3134)	7.48 (4, .1128)
Herpes; simplex virus		**1.1522 (0.95–1.40)**	**152.13 (32, <.0001)**
Van Wagoner	6.24 (df = 2) (.0442)	1.1293 (0.93–1.38)	145.89 (31, <.0001)
Laumann	4.76 (.0291)	1.1771 (0.97–1.43)	147.37 (31, <.0001)
Ferris	5.68 (.0172)	1.1735 (0.96–1.43)	146.45 (31, <.0001)
Richters	13.61 (.0002)	1.1761 (0.97–1.27)	138.52 (31, <.0001)
Schneider	26.18 (<.0001)	1.1851 (0.98–1.43)	1.25.95 (31, <.0001)
Mwandi	86.28 (<.0001)	1.0944 (0.94–1.27)	65.85 (31, .0003)
Mwandi and Schneider	99.97 (df = 2) (<.0001)	1.1311 (0.98–1.30)	51.16 (30, .0073)
Genital Warts		**0.8275 (0.65–1.04)**	**37.07 (14, .0007)**
Oriel	5.46 (.0194)	0.7792 (0.69–0.98)	31.61 (13, .0027)
Wilson	6.76 (.0093)	0.7885 (0.64–0.98)	30.31 (13, .0042)
Cook	11.89 (.0006)	0.8696 (0.70–1.08)	25.18 (13, .0218)
Cook and Wilson	18.13 (df = 2) (.0001)	0.8365 (0.69–1.01)	18.93 (12, .0901)
HPV any		**1.2411 (1.03–1.51)**	**39.98 (24, .0215)**
Lajous	4.09 (.0431)	1.1962 (0.98–1.45)	35.89 (23, .0424)
Vardas	5.56 (.0184)	1.2912 (1.05–1.59)	34.42 (23, .0593)
Vaccarella	8.29 (.0040)	1.1806 (0.99–1.40)	31.69 (23, .1068)
Vaccarella and Vardas	12.77 (df = 2) (.0017)	1.2320 (1.02–1.48)	27.21 (22, .2033)
HPV high risk		**1.1611 (0.94–1.45)**	**45.27 (24, .0054)**
Oglivie	4.01 (.0451)	1.2162 (0.98–1.51)	41.26 (23, .0111)
Svare	4.02 (.0450)	1.1323 (0.92–1.40)	41.25 (23, .0111)
Lajous	4.86 (.0274)	1.1192 (0.90–1.39)	40.41 (23, .0138)
Vardas	8.04 (.0046)	1.2186 (0.98–1.52)	37.23 (23, .0307)
Vaccarella	8.77 (.0031)	1.1049 (0.91–1.35)	36.50 (23, .0366)
Vardas and Vaccarella	15.74 (df = 2) (.0004)	1.1602 (0.95–1.41)	29.53 (22, .1303)
HPV high risk selective		**1.0128 (0.80–1.28)**	**28.82 (15, .0164)**
Aynaud et al. [33]	4.62 (.0316)	0.9727 (0.76–1.25)	24.20 (14, .0433)
Vardas	4.20 (.0404)	1.0564 (0.82–1.37)	24.62 (14, .0385)
Vaccarella	9.92 (.0016)	0.9553 (0.79–1.15)	18.90 (14, .1689)
Vaccarella and Aynaud I	15.60 (df = 2) (.0004)	0.8747 (0.74–1.03)	13.22 (13, .4306)
Vaccarella and Vardas	13.13 (df = 2) (.0014)	1.0073 (0.82–1.23)	15.69 (13, .2664)
Any STI		**0.8627 (0.7368–1.0102)**	**303.00 (26, <.0001)**
Auvert	4.55 (.0329)	0.8425 (0.7182–0.9884)	298.45 (25, <.0001)
Seed	5.63 (.0177)	0.8732 (0.7432–1.0266)	297.37 (25, <.0001)
Uganda	5.87 (.0154)	0.8410 (0.7164–0.9873)	297.13 (25, <.0001)
Urassa	15.68 (df = 5) (.0078)	0.8846 (0.7379–1.0605)	287.32 (21, <.0001)
Gebremedhin	10.48 (.0012)	0.8638 (0.7186–1.0383)	292.52 (25, <.0001)
Richters	29.28 (<.0001)	0.8713 (0.7394–1.0268)	273.72 (25, <.0001)
Ferris	35.50 (<.0001)	0.8822 (0.7542–1.0319)	2267.50 (25, <.0001)
Harbertson	49.14 (<.0001)	0.8923 (0.7665–1.0389)	253.86 (25, <.0001)
Langeni	203.41 (<.0001)	0.8248 (0.7358–0.9245)	99.59 (25, <.0001)
Langeni and Ferris	221.08 (df = 2) (<.0001)	0.8442 (0.7554–0.9434)	81.92 (24, <.0001)
Langeni and Harbertson	234.61 (df = 2) (<.0001)	0.8519 (0.7700–0.9426)	68.39 (24, <.0001)
Any STI without Langeni		**0.8248 (0.74–0.92)**	**99.59 (25, <.0001)**
Richters	5.92 (.0150)	0.8344 (0.7374–0.9442)	93.67 (24, <.0001)
Gebremedhin	7.28 (.0070)	0.8250 (0.7236–0.9406)	92.31 (24, <.0001)
Auvert	7.32 (.0068)	0.8078 (0.7219–0.9039)	92.27 (24, <.0001)
Parker	9.79 (.0017)	0.8071 (0.7195–0.9053)	89.80 (24, <.0001)
Burundi	10.37 (.0013)	0.8030 (0.7182–0.8978)	89.22 (24, <.0001)
Ferris	17.67 (<.0001)	0.8442 (0.7554–0.9434)	81.92 (24, <.0001)
Harbertson	31.20 (<.0001)	0.8519 (0.7700–0.9426)	68.39 (24, <.0001)
Harbertson and Burundi	40.87 (df = 2) (<.0001)	0.8317 (0.7544–0.9157)	58.72 (23, <.0001)
Harbertson and Ferris	51.08 (df = 2) (<.0001)	0.8761 (0.794–0.9602)	48.51 (23, .0014)

**Table 16:** Sensitivity analysis of high-risk and general populations of studies of the association between circumcision status and various sexually transmitted infections.

	Random-effects odds ratio	95% confidence interval	Heterogeneity chi-square (df)
Genital discharge syndrome			
High-risk populations	1.18	1.11–1.25	2.14 (3) *P* = .5444
General populations	0.77	0.59–0.99	16.78 (5) *P* = .0049
NSU			
High-risk populations	0.95	0.73–1.55	13.90 (6) *P* = .0308
General populations	0.61	0.48–0.76	11.76 (4) *P* = .0192
Chlamydia			
High-risk populations	1.02	0.83–1.26	9.49 (6) *P* = .1478
General populations	0.77	0.46–1.31	22.40 (6) *P* = .0010
Gonorrhea			
High-risk populations	1.09	0.86–1.40	77.07 (15) *P* < .0001
General populations	1.02	0.88–1.18	11.12 (10) *P* = .3482
GUD			
High-risk populations	1.91	1.50–2.43	15.63 (6) *P* = .0159
General populations	1.34	1.13–1.59	3.78 (4) *P* = .4371
Syphilis			
High-risk populations	1.40	1.06–1.85	39.45 (12) *P* < .0001
General populations	1.22	1.00–1.49	27.96(15) *P* = .0218
Herpes simplex			
High-risk populations	1.20	0.99–1.46	23.15 (14) *P* = .0579
General population	1.06	0.78–1.45	124.28 (17) *P* < .0001
Genital warts			
High-risk populations	0.91	0.58–1.44	28.16 (7) *P* = .0002
General populations	0.78	0.63–0.96	8.61 (6) *P* = .1969
Any HPV			
High-risk populations	1.24	0.85–1.82	14.13 (8) *P* = .0786
General populations	1.23	0.97–1.55	24.10 (15) *P* = .0634
High-risk HPV			
High-risk populations	1.08	0.72–1.63	15.82 (8) *P* = .0450
General populations	1.21	0.92–1.58	28.65 (15) *P* = .0178
Any STD			
High-risk populations	0.96	0.79–1.17	6.29(4) *P* = .1788
General populations	0.84	0.69–1.02	296.71 (20) *P* ≤ .0001
General populations; no Langerin	0.79	0.69–0.90	43.59 (19) *P* < .0001

**Table 17:** Evaluation of publication bias in studies evaluating the association between sexually transmitted diseases and circumcision status in adult males using the methods described by Egger et al. [39], Macaskill et al. [40], and Begg and Mazumdar [41].

	Begg	Begg's alternative	Egger	Egger's weighted	Macaskill	Macaskill's pooled variance
Genital discharge syndrome	0.9287	0.3252	0.0213	0.0056	0.0127	0.0171
Nonspecific urethritis	0.0549	0.0397	0.1301	0.3893	0.1322	0.0917
Chlamydia	0.8695	0.9563	0.0855	0.0003	0.1172	0.2961
Gonorrhea	0.2801	0.0404	0.3403	0.5653	0.1124	0.1764
Genital ulcerative disease	0.6547	0.7884	0.3795	0.1073	0.1804	0.3424
Syphilis	0.7356	0.6258	0.1429	0.8972	0.1023	0.6316
Genital herpes	0.2646	0.0137	0.1627	0.0014	0.3803	0.1324
Genital warts	0.5862	0.6918	0.1782	0.9378	0.5768	0.7383
Human papillomavirus: any type	0.9627	0.5592	0.1461	0.0035	0.0639	0.0857
Human papillomavirus: oncogenic type	0.8153	0.5911	0.2465	0.0889	0.0531	0.0913
Any sexually transmitted infection	0.0913	0.3482	0.1286	0.0900	<0.0001	<0.0001
Any sexually transmitted infection: without Langeni	0.8084	0.7079	0.9552	0.0765	0.2035	0.3550
